# Counselling interventions to enable women to initiate and continue breastfeeding: a systematic review and meta-analysis

**DOI:** 10.1186/s13006-019-0235-8

**Published:** 2019-10-21

**Authors:** Alison McFadden, Lindsay Siebelt, Joyce L. Marshall, Anna Gavine, Lisa-Christine Girard, Andrew Symon, Stephen MacGillivray

**Affiliations:** 1 School of Nursing and Health Sciences, University of Dundee, 11 Airlie Place, Dundee, DD1 4HJ Scotland; 2 School of Human and Health Sciences, Harold Wilson Building, University of Huddersfield, Queensgate, Huddersfield, HD1 3DH Scotland; 3 School of Health in Social Science, The University of Edinburgh, Doorway 6, Room 1m04, Old Medical School, Edinburgh, EH8 9AG Scotland

**Keywords:** Breastfeeding, Counselling, Intervention, Randomised control trial, Systematic review, Meta-analysis

## Abstract

**Background:**

Many infants worldwide are not breastfeeding according to WHO recommendations and this impacts on the health of women and children. Increasing breastfeeding is identified as a priority area supported by current policy targets. However, interventions are complex and multi-component and it is unclear which elements of interventions are most effective to increase breastfeeding in which settings. Breastfeeding counselling is often part of complex interventions but evidence is lacking on the specific effect of counselling interventions on breastfeeding practices. The aim of this systematic review is to examine evidence on effectiveness of breastfeeding counselling to inform global guidelines.

**Methods:**

A systematic search was conducted of six electronic databases in January 2018. Randomised controlled trials comparing breastfeeding counselling with no breastfeeding counselling or different formulations of counselling were included if they measured breastfeeding practices between birth and 24 months after birth.

**Results:**

From the 5180 records identified in searches and a further 11 records found by hand searching, 63 studies were included. Of these, 48 were individually-randomised trials and 15 were cluster-randomised trials. A total of 69 relevant comparisons were reported involving 33,073 women. There was a significant effect of counselling interventions on any breastfeeding at 4 to 6 weeks (Relative risk [RR] 0.85, 95% CI 0.77, 0.94) and 6 months (RR 0.92, 95% CI 0.87, 0.94). Greater effects were found on exclusive breastfeeding at 4 to 6 weeks (RR 0.79, 95% CI 0.72, 0.87) and 6 months (RR 0.84, 95% CI 0.78, 0.91). Counselling delivered at least four times postnatally is more effective than counselling delivered antenatally only and/or fewer than four times. Evidence was mostly of low quality due to high or unclear risk of bias of the included trials and high heterogeneity.

**Conclusions:**

Breastfeeding counselling is an effective public health intervention to increase rates of any and exclusive breastfeeding. Breastfeeding counselling should be provided face-to-face, and in addition, may be provided by telephone, both antenatally and postnatally, to all pregnant women and mothers with young children. To inform scale-up globally there is a need to further understand the elements of breastfeeding interventions such as counselling and their effectiveness in different contexts and circumstances.

**Study registration:**

This systematic review was registered in Prospero (CRD42018086494).

## Background

From a global perspective, the prevalence of breastfeeding varies widely, with high-income countries continually faring worse than middle- and low-income countries on nearly every standard breastfeeding indicator (i.e., from ‘ever breastfed’ to ‘breastfeeding at 12 months’ [1]). It has been estimated that infant mortality rates are nearly 12% higher when infants are not breastfed due to infections and illnesses such as pneumonia and diarrhoea [2], therefore breastfeeding has the largest known impact of any preventive intervention [3]. Additionally, the impact of breastfeeding, particularly exclusive breastfeeding, on an infant’s healthy growth and development have been well documented and not breastfeeding increases several conditions, including gastroenteritis, respiratory tract infections, obesity, and neurodevelopmental behavioural problems [4–6]. Mothers who do not breastfeed also have increased risks of breast and ovarian cancer, obesity, type II diabetes and postpartum depression [6–8]. It is therefore not surprising that exclusive breastfeeding has been identified as a priority area, with global targets recently increased from 50% of children being exclusively breastfed at 6 months by 2025 to at least 70% by 2030 [9]. A call for scaling up programming efforts to achieve these goals has been put forward, yet the question remains as to which elements of past and current preventative intervention programming are most effective in increasing standard breastfeeding indicators.

Both design and implementation of public health preventive interventions targeting breastfeeding indicators are vastly heterogeneous and often multi-component, making the ability to concisely evaluate which specific elements are most effective challenging. For example, preventative interventions vary in type (e.g., counselling, education, Baby-Friendly Hospital Initiative (BFHI), support, media and mass-marketing), setting (e.g., hospital, health facility, community/home), mode (e.g., group, individual, telephone, face-to-face), provider (e.g., healthcare professional, layperson/peer) stage of delivery (e.g., antenatal, postpartum), and frequency. Moreover, consensus and standard definitions, particularly related to types of preventative interventions, are lacking and often ill-defined. Recent systematic reviews have aimed to evaluate the efficacy that differing programming efforts have had in increasing standard breastfeeding indicators [10–15]. While these reviews all report effectiveness of interventions to increase breastfeeding rates, the effect size varies dependant on the review inclusion criteria and outcomes assessed.

While these reviews have advanced our knowledge regarding evaluation of best practices, gaps in the knowledge base remain. This is in part due to considerable heterogeneity in interventions and outcomes, and is compounded by poor reporting on/definitions of the differing ‘types’ of interventions. A systematic review by Sinha et al. [14], found that interventions that are complex and delivered in a combination of settings (e.g. interventions involving health systems, such as the BFHI were most effective). However, it is difficult to identify which elements of such complex, multi-component interventions are effective in which settings. Breastfeeding counselling is often part of complex interventions but evidence is lacking on the specific effect of counselling interventions on breastfeeding practices.

Barriers in operationalising what counselling specifically entails is a consistent shortcoming in published studies and protocols. When the term ‘counselling’ is used there is often much overlap with other types of preventative interventions such as education, resulting in difficultly in differentiating between the two. The World Health Organization (WHO) defines breastfeeding counselling as the support of mothers and infants, as provided by healthcare workers, in decision-making, overcoming difficulties, and implementation of optimal feeding practices [16, 17]. A key element is the *interaction* that takes places between a healthcare worker and mother, which should support women and their decision making. Counselling is therefore a type of preventative intervention which places emphasis on the dyadic interaction between a healthcare worker and a mother, rather than the top-down approach often more characteristic of education-based types of interventions. Counselling is therefore a type of support delivered directly to mothers and infants. All counselling can be considered support but not all support interventions involve counselling. For example, this review does not include studies of only higher-level interventions such as additional training for staff providing support [18–20] or policy interventions [21].

The aim of this systematic review and meta-analysis was to examine the evidence on the effectiveness of breastfeeding counselling to inform global guidelines [22].

## Methods

This systematic review followed the Cochrane Collaboration guidance [23], and was registered in Prospero (CRD42018086494).

### Search strategy

We searched six electronic databases: WHO International Clinical Trials Registry; clinicaltrials.gov; Cochrane Trials Register, Medline, CINAHL and Embase in January 2018, using the key search terms “breastfeeding” AND “counselling” AND “trials” (see Additional file 1 for detailed search strategy in Medline). We did not apply any language or date restrictions. We also scanned reference lists of three relevant systematic reviews [11, 14, 15]. Title, abstracts and potentially relevant full texts were screened independently by two authors and any disagreement resolved through discussion and consultation with a third author.

### Inclusion criteria

Studies were included if they were randomised (including cluster-randomised, or quasi-randomised controlled), involving pregnant women considering or intending to breastfeed, or women who may initiate or are breastfeeding. Eligible interventions were those described as (or containing elements of) breastfeeding counselling. For the purposes of this review breastfeeding counselling was defined as a process by which a health worker supports mothers and infants to implement optimal feeding practices and helps them to overcome difficulties, involving interaction with a woman to support her in solving actual or anticipated problems, reviewing options, and making decisions. Interventions described as ‘counselling’ but where insufficient detail was reported to judge whether it met the above definition were included, as were interventions described as education or home visits that included features of counselling such as discussion of breastfeeding goals, challenges and techniques. Eligible comparisons were no breastfeeding counselling or different formulations of counselling. To be included, studies had to report at least one of the following outcomes:

- Number of women who do not initiate breastfeeding within 1 h of birth;
- Number of women who stop any breastfeeding before 6 months as assessed at two time-points:
  - ◦ Four to six weeks postpartum;
  - ◦ Six months postpartum;
- Number of women who stop exclusive breastfeeding before 6 months as assessed at two time-points:
  - ◦ Four to six weeks postpartum;
  - ◦ Six months postpartum;
- Number of women who stop any breastfeeding before 12 months postpartum;
- Number of women who stop any breastfeeding before 24 months postpartum;
- Number of newborns given prelacteal or additional food, fluids or infant formula within the first 3 days postpartum;
- Number of infants fed with bottles during the first 6 months postpartum. For this outcome we included studies that reported the number of infants fed with bottles at the last study assessment.

### Exclusion criteria

Non-randomised designs were excluded as were interventions targeted only at families, communities or healthcare providers. We excluded interventions that did not include any elements of the above definition of breastfeeding counselling, and multi-component interventions where the effects could not be attributed only to counselling.

### Data extraction, risk of bias and quality of evidence assessment

Two authors independently extracted information using a specifically designed data extraction form. Any discrepancies were resolved through discussion. When information regarding study methods and results was unclear, we attempted to contact authors to provide further details. Two authors independently assessed risk of bias for each study using the criteria outlined in the Cochrane Handbook for Systematic Reviews of Interventions [23]. Any disagreement was resolved by discussion with a third assessor. We used the GRADE approach [24] to assess the quality of the evidence.

### Data analysis and synthesis

Cluster randomised trial sample sizes were adjusted, incorporating an estimate of the intra-cluster correlation coefficient (ICC) derived from the trial (if possible) to calculate an effective sample size. To avoid ‘double counting’ in multi-arm studies, we split the control group number of events and participants in half to enable two independent comparisons. For all outcomes, analyses were carried out, where possible, on an intention-to-treat basis. The denominator for each outcome in each trial was the number randomised minus any participants whose outcomes were known to be missing.

We carried out statistical analysis using Review Manager 5 software [25]. We used random-effects meta-analysis for combining data where significant statistical heterogeneity was present. The average treatment effects with 95% confidence intervals are presented.

### Subgroup analysis

Where data were available, we carried out the following seven main subgroup analyses for all review outcomes: timing, frequency, mode, provider of counselling, involvement of anticipatory approaches, setting, and counselling targeted for specific population sub-groups.

### Sensitivity analysis

We carried out sensitivity analysis for all outcomes by study quality by dividing the studies according to whether they were at low risk of bias as opposed to unclear or high risk of bias for allocation concealment.

## Results

### Results of the search

**Fig. 1:**
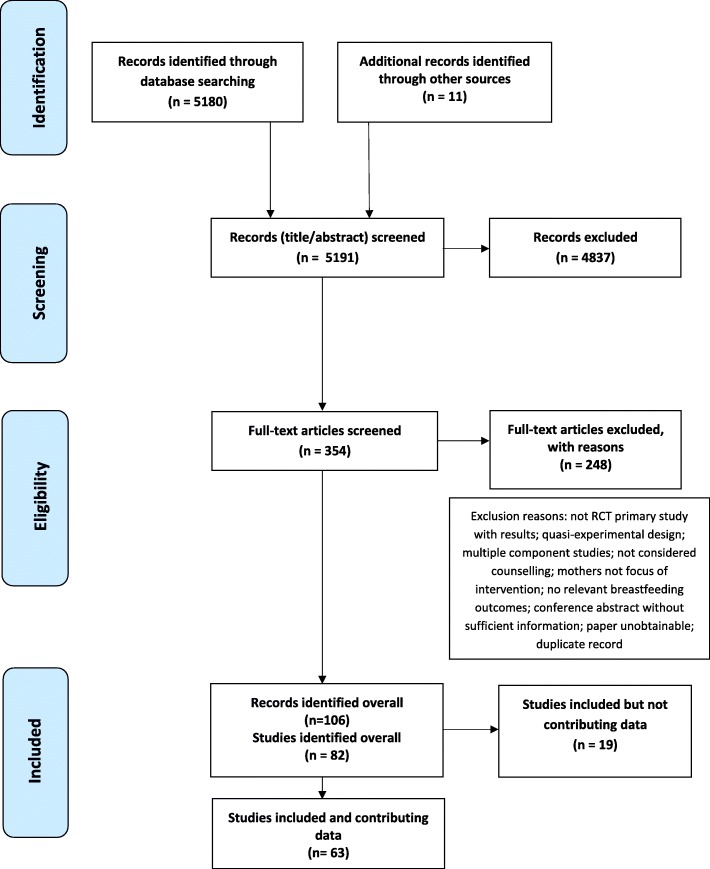
Preferred reporting items for systematic reviews and meta-analyses (PRISMA) flow chart

The searches resulted in 5180 original records and a further 11 records were identified through checking reference lists of included studies and published systematic reviews. We excluded 4837 titles and abstracts. We assessed 354 full text articles and excluded 248, leaving 106 articles reporting 82 studies (see Fig. 1). Nineteen studies were subsequently excluded from the analyses because they did not report data in a useable form or did not report relevant outcomes [26–44]. Therefore 63 studies contributed data to the analyses (see Fig. 1). Of note, two trials were published in a single paper [45]. To differentiate we refer to the BINGO trial as Bonuck (BINGO) [45] and the PAIRINGS trial as Bonuck (PAIRINGS) [45]. Additionally, the PROMISE-EBF trial [46] was conducted in three countries which we have classed as three separate trials due to substantial differences between countries in the trial methods and comparators. The 63 studies comprised 48 individually-randomised and 15 cluster-randomised trials. The 63 trials incorporated 69 comparisons as six studies [47–52] had two relevant interventions in different trial arms. These are referred to separately by the author name and the relevant feature of the trial arm (e.g. home based or facility based). More specifically, Aidam AN & PN [47] refers to counselling provided in the antenatal and postnatal period and Aidam PN [47] refers to counselling provided in the postnatal period only. Fu Hospital [48] refers to in-hospital visits only and Fu Telephone refers to telephone calls only. McLachlan [49] Home refers to a home visiting intervention and McLachlan Drop-in refers to home visiting plus access to a community-based breastfeeding drop-in centre. Morrow [50] three visits and Morrow six visits refer to the number of visits in different intervention arms. Ochola [51] Facility refers to counselling delivered in a healthcentre and Ochola Home refers to counselling delivered in the home. Su [52] AN refers to counselling delivered in the antenatal period only and Su PN refers to counselling delivered in the postnatal period only. See Additional file 2 for further details of all included studies (*n* = 63).

### Participants and settings

The participants in the 63 trials comprised 33,073 women and their infants (range 41–9675) from 26 countries. Almost two thirds of the studies, representing 76% of the participants, were from 10 high-income countries (40 studies; 25,223 participants). Fourteen studies, involving 3236 participants (10% of participants) were from nine upper middle-income countries. Seven studies involving 3055 participants (9% of participants) were from five lower middle-income countries. Two studies, both from the PROMISE-EBF trial [46], involving 1559 participants (5% of the total number of participants) were from two low-income countries, Burkina Faso and Uganda.

The 63 studies encompassed a broad range of participant characteristics. For example, mean age was most commonly in the 20 to 30 year range (32 studies) with eight studies having a mean of over 30 years and just one study of women under 20 years [53]. In the remaining studies age was unclear or not reported.

Two thirds (*n* = 42) of the studies included both primiparous and multiparous women, while seven included only primiparous women and seven studies did not report parity. Four studies excluded women who had caesarean births [54–57], while the remaining studies either included both vaginal and caesarean births or did not report mode of birth. Most studies included only healthy term newborns with no congenital anomalies or admission to neonatal unit. One study recruited only low birthweight infants [56], another recruited only preterm infants [58], one study was of twin births only [59] and another included only those who were jaundiced [60].

### Interventions

A broad range of interventions was apparent across the 63 studies. The amount and nature of counselling along with detail of interventions reported varied considerably. Interventions commonly included an element of education, for example, about the importance and benefits to health of exclusive breastfeeding, but women were usually also encouraged to ask questions and raise concerns [47, 48, 56, 61, 62]. Many interventions included the provision of technical information and support for practical aspects of breastfeeding, for example giving advice or ensuring the baby was breastfeeding effectively e.g. [50, 63–68] or counselling to manage problems [57, 69, 70].

The timing, frequency and intensity of contacts varied considerably across the 69 interventions. Most interventions were provided only after birth (*n* = 35) or had antenatal and postnatal components (*n* = 26). The majority of postnatal interventions (whether there was an antenatal component or not) included at least one contact during the first 6 days following birth (*n* = 43). Eight interventions were provided only in the antenatal period and one study did not clearly report when the intervention was provided [71]. The number of sessions ranged from one to 24 scheduled contacts with over half of the interventions including more than four contacts (*n* = 39).

More than two thirds of interventions were provided to women one-to-one (*n* = 51) whereas some interventions included one-to-one and group contacts (*n* = 7). Sessions were less often provided only to groups of women (*n* = 3) [59, 72, 73] and one study involved two supporters for each mother [57]. The majority of interventions were provided face-to-face (*n* = 37) but many also incorporated counselling by telephone (*n* = 28) and three interventions were delivered by telephone only [55, 74, 75]. Interventions were provided by a range of personnel and volunteers and were categorized into lay (peer, community health workers, doulas) or non-lay (health professionals, lactation consultants/counsellors, breastfeeding consultants/counsellors and researchers). In several trials members of the research team delivered the intervention, and it was not always clear whether these personnel were health professionals. In many studies specific training was provided to counsellors but this was often poorly reported. When reported, the content and length of the training were variable (e.g. some had no training; brief orientation; between 6 and 40 h or 2 weeks).

### Comparisons

Most comparisons were described in the studies as usual or standard care and in ten studies this included either care in a UNICEF/WHO accredited hospital [50, 63, 66, 76, 77], working towards accreditation [78] or a session using UNICEF/WHO guidelines [57, 59, 61, 75]. In 11 studies extra contacts were provided either to support women with different aspects such as breast examination or infant safety, or to provide counselling that was not breastfeeding related such as general nutrition counselling.

### Risk of bias

Included studies were judged to be of mixed risk of bias across all domains. For example, just under half (49%) were judged to be at low risk of bias for allocation concealment; 40% were judged to be at low risk of bias for blinding of outcome assessment and 46% were judged to be of low risk of bias for incomplete outcome data. It was difficult to assess selective outcome reporting because so few studies referred to a registered/published protocol. All studies were judged to be at high or unclear risk of performance bias as it is not possible to blind participants and personnel to counselling interventions.

### Effects of interventions

#### Counselling interventions compared to no counselling/standard care

**Fig. 2:**
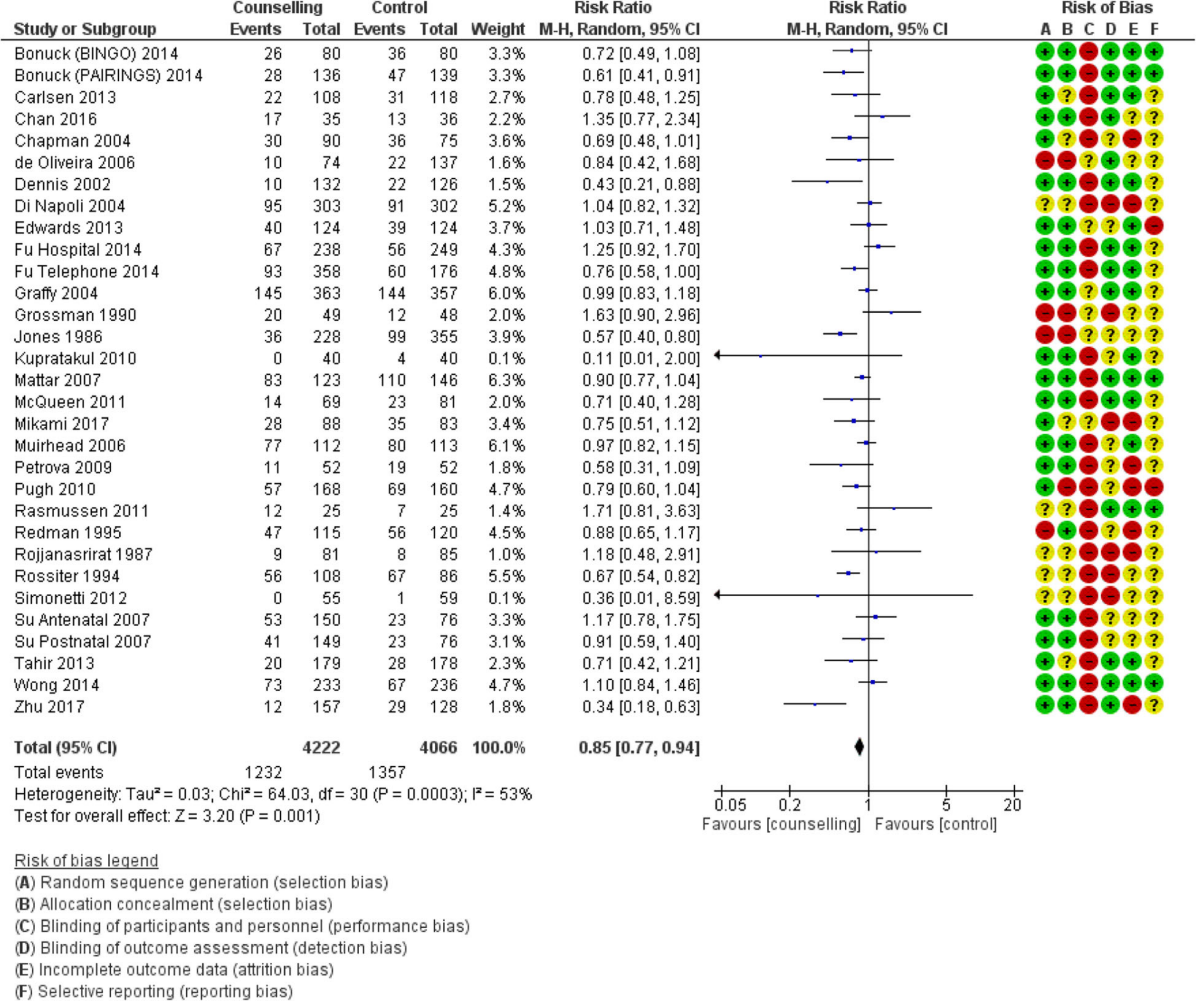
Number of women who stop any breastfeeding before 4–6 weeks postpartum

**Fig. 3:**
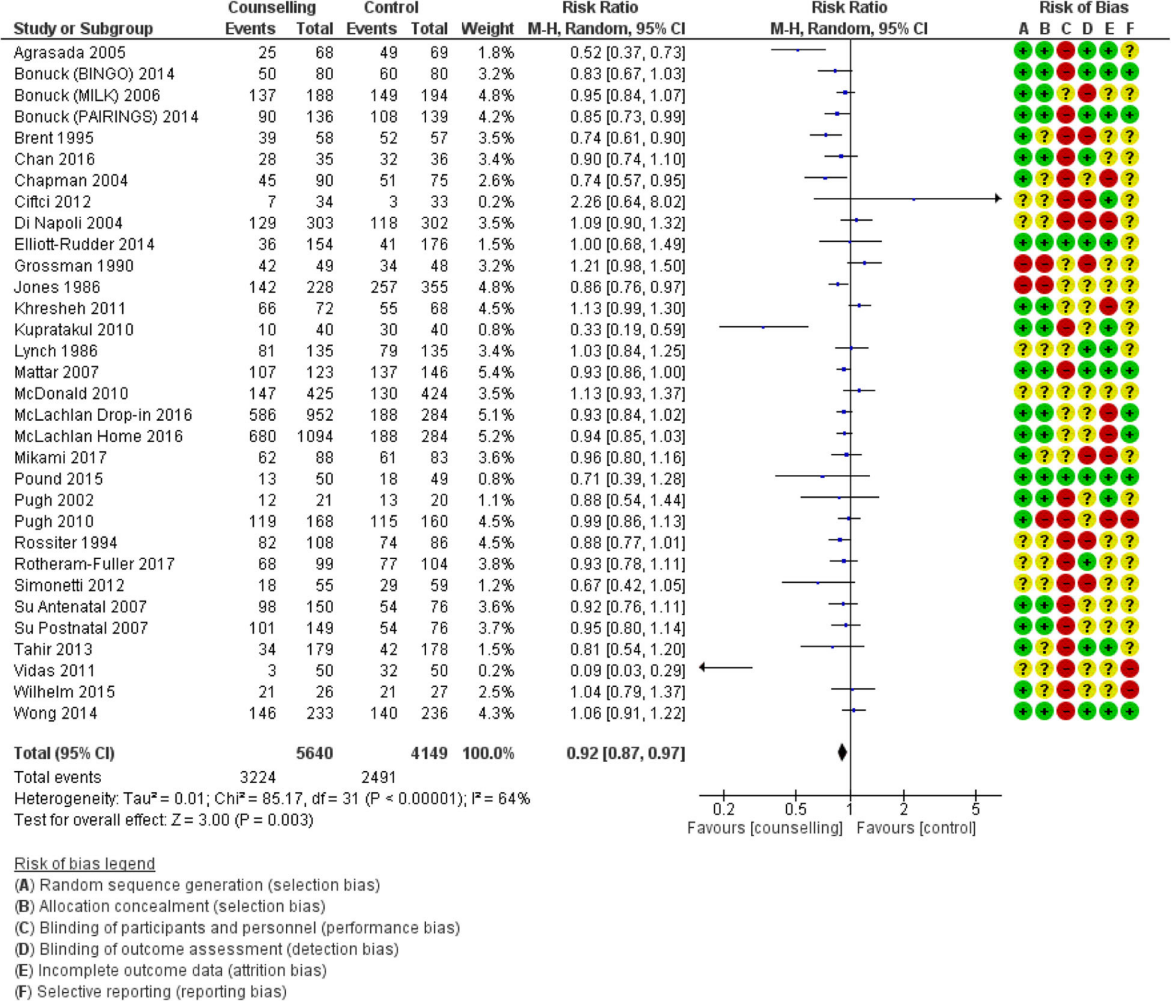
Number of women who stop any breastfeeding before 6 months postpartum

**Fig. 4:**
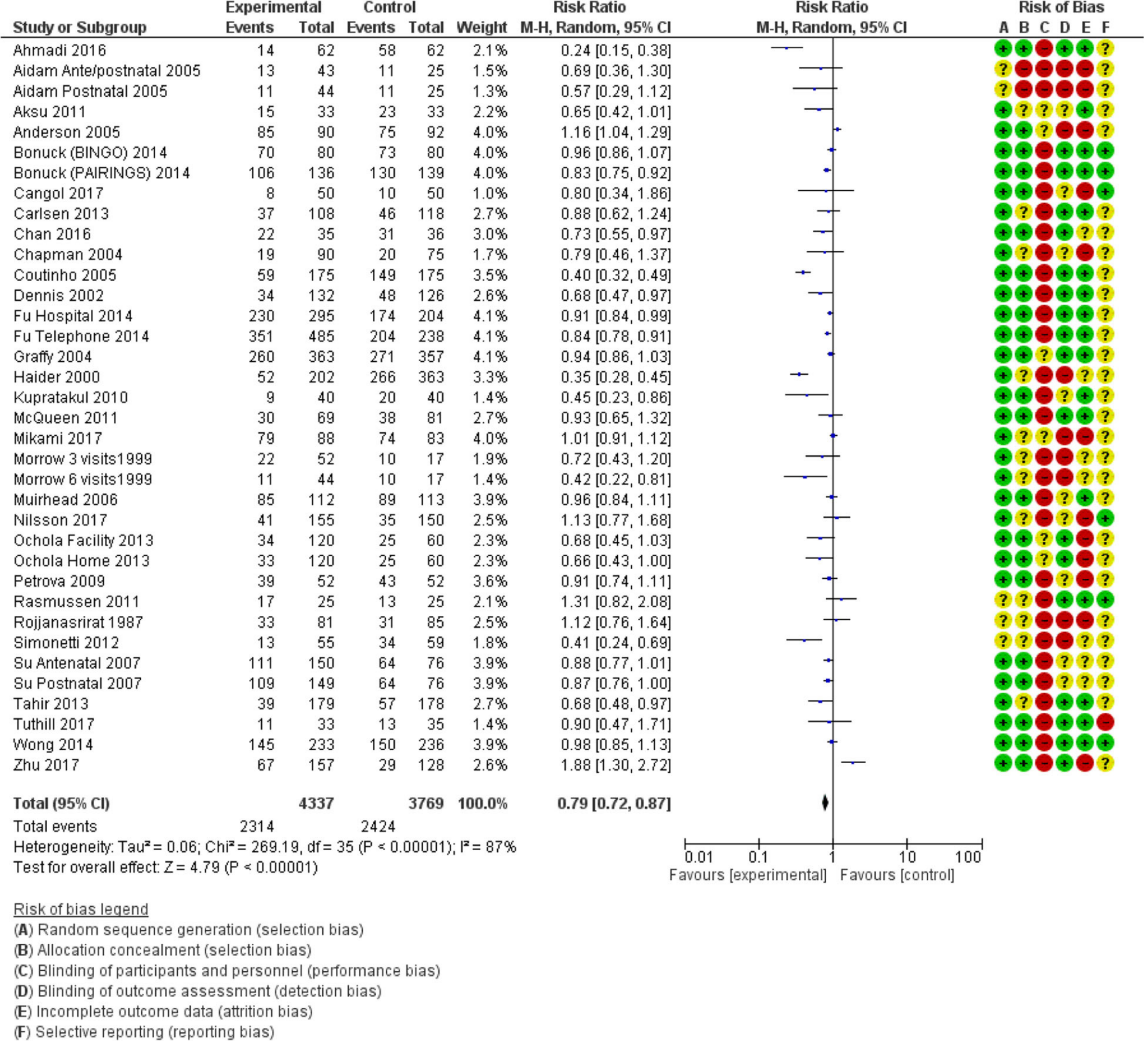
Number of women who stop exclusive breastfeeding before 4–6 weeks postpartum

**Fig. 5:**
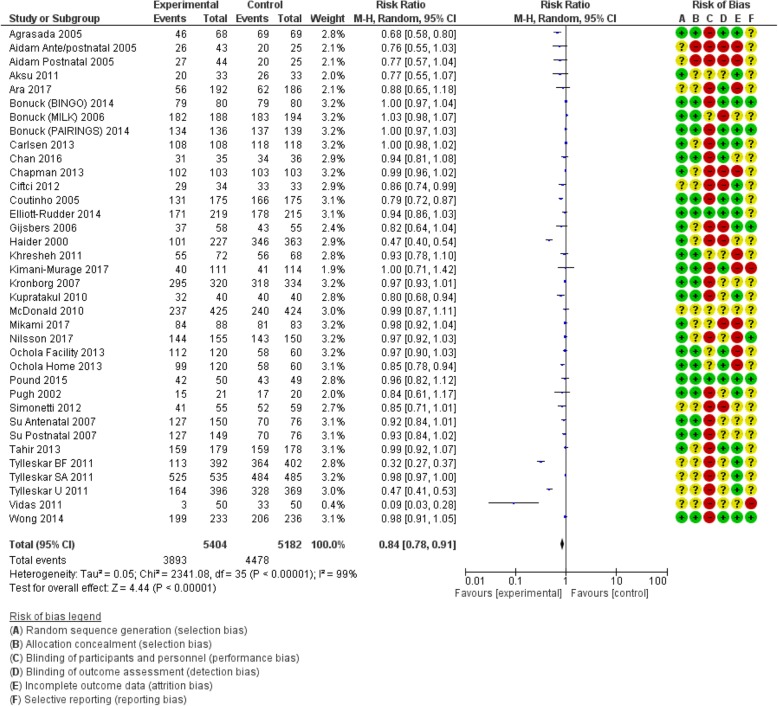
Number of women who stop exclusive breastfeeding before 6 months postpartum

Table 1 shows the pooled effects of counselling interventions on eight of the nine outcomes of this review; no trials assessed breastfeeding rates at 24 months. Counselling interventions reduced the risk of women stopping any breastfeeding at 4 to 6 weeks by 15% (risk ratio [RR] 0.85, 95% confidence interval [CI] 0.77,0.94), and at 6 months by 8% (RR 0.92, CI 0.87,0.97). For exclusive breastfeeding the effect was greater with a 21% (RR 0.79, CI 0.72, 0.87) reduction at 4 to 6 weeks and a 16% (RR 0.84, CI 0.78, 0.91) reduction at 6 months. Sensitivity analyses demonstrated a similar positive effect. Statistical heterogeneity was significant for all outcomes: any breastfeeding at 4 to 6 weeks Tau^2^ = 0.03, I^2^ = 53%, Chi^2^ = 64.03, *p* < 0.0003); any breastfeeding at 6 months Tau^2^ = 0.01, I^2^ = 64%, Chi^2^ = 85.17, *p* < 0.00001; exclusive breastfeeding at 4 to 6 weeks (Tau^2^ = 0.06, I^2^ = 87%, Chi^2^ = 269.19, *p* < 0.00001); exclusive breastfeeding at 6 months (Tau^2^ = 0.05, I^2^ = 99%, Chi^2^ = 2341.08, *p* < 0.00001). The quality of the evidence was low due to high or unclear risk of bias and high unexplained heterogeneity (Figs. 2, 3, 4 and 5).

**Table 1:** Effects of counselling versus no counselling

Number of trials	No of events		Effect		Certainty
	Intervention	Control	Relative (95% CI)	Absolute (95% CI)	
Number of women who do not initiate breastfeeding within 1 h of birth					
7 trials	1038/1913 (54.3%)	1188/1818 (65.3%)	RR 0.74 (0.53 to 1.02)	170 fewer per 1000 (from 13 more to 307 fewer)	Moderate
Number of women who stop any breastfeeding before 4–6 weeks postpartum					
29 trials (31 comparisons)	1232/4222 (29.2%)	1357/4066 (33.4%)	RR 0.85 (0.77 to 0.94)	50 fewer per 1000 (from 20 fewer to 77 fewer)	Low
Number of women who stop any breastfeeding before 6 months postpartum					
30 trials (32 comparisons)	3224/5640 (57.2%)	2491/4149 (60.0%)	RR 0.92 (0.87 to 0.97)	48 fewer per 1000 (from 18 fewer to 78 fewer)	Low
Number of women who stop exclusive breastfeeding before 4–6 weeks postpartum					
31 trials (36 comparisons)	2314/4337 (53.4%)	2424/3769 (64.3%)	RR 0.79 (0.72 to 0.87)	135 fewer per 1000 (from 84 fewer to 180 fewer)	Low
Number of women who stop exclusive breastfeeding before 6 months postpartum					
33 trials (36 comparisons)	3893/5404 (72.0%)	4478/5182 (86.4%)	RR 0.84 (0.78 to 0.91)	138 fewer per 1000 (from 78 fewer to 190 fewer)	Low
Number of women who stop any breastfeeding before 12 months postpartum					
2 trials	349/416 (83.9%)	516/549 (94.0%)	RR 0.88 (0.69 to 1.12)	113 fewer per 1000 (from 113 more to 291 fewer)	Low
Number of newborns given prelacteal or additional food, fluids or infant formula within the first 2 days postpartum					
1 trial	26/50 (52.0%)	40/50 (80.0%)	RR 0.65 (0.48 to 0.88)	280 fewer per 1000 (from 96 fewer to 416 fewer)	Low
Number of infants fed with bottles during the first 6 months postpartum					
5 trials	349/416 (83.9%)	516/549 (94.0%)	RR 0.88 (0.69 to 1.12)	113 fewer per 1000 (from 113 more to 291 fewer)	Moderate

The pooled effects of counselling interventions on the number of women who did not initiate breastfeeding within 1 h of birth, the number of women who stopped breastfeeding at 12 months and the number of infants fed with bottles during the first 6 months were not statistically significant. One trial of 100 women [79] provided moderate quality evidence that counselling interventions reduced the number of infants given prelacteal or other additional food, fluids or infant formula in the first 3 days of life by 35% (RR 0.65, CI 0.48,0.88).

#### Sub-group analyses: intervention characteristics

Because of the low number of relevant trials, we did not conduct sub-group analyses for two outcomes: number of women who stop breastfeeding at 12 months (two trials), and number of newborns given prelacteal or other foods or fluids within the first 2 days following birth (one trial).

##### Timing

Table 2 shows the effects of counselling interventions according to whether the intervention was provided antenatally, postnatally, or both. For ‘any breastfeeding’, six trials provided low quality evidence that counselling interventions delivered both antenatally and postnatally were more effective in reducing the risk of women stopping any breastfeeding before 6 months (21% reduction, RR 0.79, CI 0.67,0.93) compared to those provided only in the antenatal or postnatal periods. For exclusive breastfeeding at both time points, counselling interventions with a postnatal component were more effective than counselling delivered antenatally only. For example, based on 15 trials involving 5411 women, counselling interventions with antenatal and postnatal components reduced the risk of women stopping exclusive breastfeeding before 6 months by 29% (RR 0.71, CI 0.55, 0.93). The quality of evidence was low due to high or unclear risk of bias and high unexplained heterogeneity. One trial [72] provided evidence that counselling in the antenatal period only reduced the risk of women not initiating breastfeeding within 1 h of birth although this trial was at high risk of bias. Moderate evidence from three trials (659 women) suggested that counselling interventions provided postnatally only reduce the risk of infants being fed with bottles during the first 6 months, by 23% (RR 0.77, CI 0.68,0.87).

**Table 2:** Effects of different timings of interventions

Time intervention delivered	Number of trials	No of events		Effect		Certainty
		Intervention	Control	Relative (95% CI)	Absolute (95% CI)	
Number of women who do not initiate breastfeeding within 1 h of birth						
Antenatal only	1 trial	35/108 (32.4%)	58/86 (67.4%)	RR 0.48 (0.35 to 0.65)	351 fewer per 1000 (from 236 fewer to 438 fewer)	Low
Antenatal and postnatal	6 trials	1003/1805 (55.6%)	1130/1732 (65.2%)	RR 0.79 (0.57 to 1.08)	137 fewer per 1000 (from 52 more to 281 fewer)	Low
Number of women who stop any breastfeeding before 4–6 weeks postpartum						
Antenatal only	6 trials	319/782 (40.8%)	338/707 (47.8%)	RR 0.86 (0.72 to 1.03)	67 fewer per 1000 (from 14 more to 134 fewer)	Low
Postnatal only	12 trials (13 comparisons)	461/2039 (22.6%)	467/1838 (25.4%)	RR 0.83 (0.69 to 1.00)	43 fewer per 1000 (from 0 fewer to 79 fewer)	Low
Antenatal and postnatal	11 trials	416/1173 (35.5%)	453/1166 (38.9%)	RR 0.91 (0.78 to 1.05)	35 fewer per 1000 (from 19 more to 85 fewer)	Moderate
Number of women who stop any breastfeeding before 6 months postpartum						
Antenatal only	6 trials	545/782 (69.7%)	526/707 (74.4%)	RR 0.93 (0.88 to 0.98)	52 fewer per 1000 (from 15 fewer to 89 fewer)	Moderate
Postnatal only	18 trials (19 comparisons)	2188/4083 (53.6%)	1286/2546 (50.5%)	RR 0.96 (0.88 to 1.04)	20 fewer per 1000 (from 20 more to 61 fewer)	Low
Antenatal and postnatal	6 trials	334/547 (61.1%)	407/541 (75.2%)	RR 0.79 (0.67 to 0.93)	158 fewer per 1000 (from 53 fewer to 248 fewer)	Low
Number of women who stop exclusive breastfeeding before 4–6 weeks postpartum						
Antenatal only	6 trials	450/704 (63.9%)	399/570 (70.0%)	RR 0.95 (0.89 to 1.02)	35 fewer per 1000 (from 14 more to 77 fewer)	Moderate
Postnatal only	12 trials (13 comparisons)	1009/1943 (51.9%)	935/1503 (62.2%)	RR 0.71 (0.59 to 0.85)	180 fewer per 1000 (from 93 fewer to 255 fewer)	Low
Antenatal and postnatal	16 trials (17 comparisons)	855/1690 (50.6%)	1090/1696 (64.3%)	RR 0.81 (0.69 to 0.94)	122 fewer per 1000 (from 39 fewer to 199 fewer)	Low
Number of women who stop exclusive breastfeeding before 6 months postpartum						
Antenatal only	5 trials	601/671 (89.6%)	494/535 (92.3%)	RR 0.98 (0.96 to 1.01)	18 fewer per 1000 (from 9 more to 37 fewer)	Moderate
Postnatal only	16 trials	1483/2002 (74.1%)	1575/1926 (81.8%)	RR 0.88 (0.81 to 0.96)	98 fewer per 1000 (from 33 fewer to 155 fewer)	Low
Antenatal and postnatal	15 trials	1663/2718 (61.2%)	2334/2693 (86.7%)	RR 0.71 (0.55 to 0.93)	251 fewer per 1000 (from 61 fewer to 390 fewer)	Low
Number of infants fed with bottles during the first 6 months postpartum						
Postnatal only	3 trials	143/331 (43.2%)	188/328 (57.3%)	RR 0.77 (0.68 to 0.87)	132 fewer per 1000 (from 75 fewer to 183 fewer)	Moderate
Antenatal and postnatal	2 trials	260/398 (65.3%)	280/393 (71.2%)	RR 0.92 (0.85 to 1.00)	57 fewer per 1000 (from 0 fewer to 107 fewer)	Moderate

##### Frequency

Table 3 shows the pooled effect of counselling interventions categorised by whether counselling was delivered fewer than four times or four or more times. Interventions delivered four or more times showed a statistically significant effect on both any and exclusive breastfeeding, but the effect size was greater for exclusive breastfeeding. The greatest effect was for exclusive breastfeeding at 4 to 6 weeks, which showed a 31% (RR 0.69, CI 0.58, 0.82) reduction in the risk of women stopping breastfeeding. The comparative reduction for the same outcome for counselling delivered on fewer than four occasions was 8% (RR 0.92, CI 0.88, 0.97). The evidence was low quality due to high or unclear risk of bias and high unexplained heterogeneity. There was high quality evidence from one trial involving 350 women [61] that counselling interventions with four or more contacts reduced the risk of infants being fed with bottles in the first 6 months by 23% (RR 0.77, CI 0.68, 0.88).

**Table 3:** Effects of different frequencies of interventions

Frequency of intervention	Number of trials	No of events		Effect		Certainty
		Intervention	Control	Relative (95% CI)	Absolute (95% CI)	
Number of women who do not initiate breastfeeding within 1 h of birth						
< 4 times	1 trial	35/108 (32.4%)	58/86 (67.4%)	RR 0.48 (0.35 to 0.65)	351 fewer per 1000 (from 236 fewer to 438 fewer)	Very low
≥ 4 times	6 trials	1003/1805 (55.6%)	1130/1732 (65.2%)	RR 0.79 (0.57 to 1.08)	137 fewer per 1000 (from 52 more to 281 fewer)	Low
Number of women who stop any breastfeeding before 4–6 weeks postpartum						
< 4 times	14 trials (15 comparisons)	733/2129 (34.4%)	725/2050 (35.4%)	RR 0.95 (0.84 to 1.07)	18 fewer per 1000 (from 25 more to 57 fewer)	Low
≥ 4 times	15 trials	463/1865 (24.8%)	533/1661 (32.1%)	RR 0.77 (0.66 to 0.90)	74 fewer per 1000 (from 32 fewer to 109 fewer)	Low
Number of women who stop any breastfeeding before 6 months postpartum						
< 4 times	13 trials (14 comparisons)	1599/2675 (59.8%)	1029/1720 (59.8%)	RR 0.96 (0.92 to 1.01)	24 fewer per 1000 (from 6 more to 48 fewer)	Moderate
≥ 4 times	16 trials	882/1785 (49.4%)	1002/1790 (56.0%)	RR 0.85 (0.75 to 0.96)	84 fewer per 1000 (from 22 fewer to 140 fewer)	Low
Number of women who stop exclusive breastfeeding before 4–6 weeks postpartum						
< 4 times	15 trials (16 comparisons)	1178/1971 (59.8%)	1036/1629 (63.6%)	RR 0.92 (0.88 to 0.97)	51 fewer per 1000 (from 19 fewer to 76 fewer)	Moderate
≥ 4 times	19 trials (20 comparisons)	1136/2366 (48.0%)	1388/2140 (64.9%)	RR 0.69 (0.58 to 0.82)	201 fewer per 1000 (from 117 fewer to 272 fewer)	Low
Number of women who stop exclusive breastfeeding before 6 months postpartum						
< 4 times	12 trials (13 comparisons)	1444/1682 (85.9%)	1326/1471 (90.1%)	RR 0.96 (0.94 to 0.98)	36 fewer per 1000 (from 18 fewer to 54 fewer)	Moderate
≥ 4 times	22 trials (23 comparisons)	2369/3709 (63.9%)	3077/3683 (83.5%)	RR 0.76 (0.66 to 0.88)	201 fewer per 1000 (from 100 fewer to 284 fewer)	Low
Number of infants fed with bottles during the first 6 months postpartum						
< 4 times	4 trials	292/554 (52.7%)	324/546 (59.3%)	RR 0.91 (0.82 to 1.01)	53 fewer per 1000 (from 6 more to 107 fewer)	Moderate
≥ 4 times	1 trial	111/175 (63.4%)	144/175 (82.3%)	RR 0.77 (0.68 to 0.88)	189 fewer per 1000 (from 99 fewer to 263 fewer)	High

##### Mode of provision

We conducted sub-group analyses according to whether the counselling was delivered face-to-face, by telephone, or included a combination of both (see Table 4). The greatest effect was again for exclusive breastfeeding at 4 to 6 weeks where there was low quality evidence from 17 comparisons (3550 women) that face-to-face counselling reduced the risk of women stopping breastfeeding by a third (RR 0.67, CI 0.56, 0.81). For this outcome there was also a statistically significant effect of counselling by telephone, which reduced the risk of women stopping exclusive breastfeeding at 4 to 6 weeks by 28% (RR 0.72, CI 0.55, 0.95). For the outcome exclusive breastfeeding at 6 months, pooled analysis of interventions delivered face-to-face showed a statistically significant effect, reducing the risk of women stopping breastfeeding by 26% (RR 0.74, CI 0.63, 0.87). The pooled effects of counselling interventions on the number of women who did not initiate breastfeeding within 1 h of birth, and the number of infants fed with bottles during the first 6 months were not statistically significant.

**Table 4:** Effects of different modes of interventions

Mode of intervention	Number of trials	No of events		Effect		Certainty
		Intervention	Control	Relative (95% CI)	Absolute (95% CI)	
Number of women who do not initiate breastfeeding within 1 h of birth						
Face-to-face	6 trials	1022/1863 (54.9%)	1167/1768 (66.0%)	RR 0.73 (0.52 to 1.03)	178 fewer per 1000 (from 20 more to 317 fewer)	Very low
Face-to-face and telephone	1 trial	16/50 (32.0%)	21/50 (42.0%)	RR 0.76 (0.45 to 1.28)	101 fewer per 1000 (from 118 more to 231 fewer)	Very low
Number of women who stop any breastfeeding before 4–6 weeks postpartum						
Face-to-face	10 trials (11 comparisons)	520/1586 (32.8%)	594/1644 (36.1%)	RR 0.86 (0.75 to 1.00)	51 fewer per 1000 (from 0 fewer to 90 fewer)	Low
Telephone	4 trials	135/700 (19.3%)	120/531 (22.6%)	RR 0.75 (0.61 to 0.93)	56 fewer per 1000 (from 16 fewer to 88 fewer)	Moderate
Face-to-face and telephone	16 trials	577/1936 (29.8%)	643/1891 (34.0%)	RR 0.86 (0.73 to 1.01)	48 fewer per 1000 (from 3 more to 92 fewer)	Low
Number of women who stop any breastfeeding before 6 months postpartum						
Face-to-face	13 trials (14 comparisons)	893/1541 (57.9%)	1001/1542 (64.9%)	RR 0.89 (0.81 to 0.98)	71 fewer per 1000 (from 13 fewer to 123 fewer)	Low
Telephone	2 trials	52/234 (22.2%)	71/237 (30.0%)	RR 0.74 (0.55 to 1.00)	78 fewer per 1000 (from 0 fewer to 135 fewer)	Moderate
Face-to-face and telephone	15 trials (16 comparisons)	2264/3865 (58.6%)	1404/2370 (59.2%)	RR 0.95 (0.88 to 1.02)	30 fewer per 1000 (from 12 more to 71 fewer)	Low
Number of women who stop exclusive breastfeeding before 4–6 weeks postpartum						
Face-to-face	13 trials (17 comparisons)	1019/1923 (53.0%)	1200/1627 (73.8%)	RR 0.67 (0.56 to 0.81)	243 fewer per 1000 (from 140 fewer to 325 fewer)	Low
Telephone	4 trials	440/827 (53.2%)	341/593 (57.5%)	RR 0.72 (0.55 to 0.95)	161 fewer per 1000 (from 29 fewer to 259 fewer)	Moderate
Face-to-face and telephone	15 trials	855/1587 (53.9%)	883/1549 (57.0%)	RR 0.96 (0.86 to 1.07)	23 fewer per 1000 (from 40 more to 80 fewer)	Low
Number of women who stop exclusive breastfeeding before 6 months postpartum						
Face-to-face	21 trials (24 comparisons)	2587/3887 (66.6%)	3196/3653 (87.5%)	RR 0.74 (0.63 to 0.87)	227 fewer per 1000 (from 114 fewer to 324 fewer)	Low
Telephone	3 trials	285/342 (83.3%)	306/355 (86.2%)	RR 0.96 (0.83 to 1.12)	34 fewer per 1000 (from 103 more to 147 fewer)	Low
Face-to-face and telephone	9 trials	875/1162 (75.3%)	901/1146 (78.6%)	RR 0.96 (0.91 to 1.01)	31 fewer per 1000 (from 8 more to 71 fewer)	Low
Number of infants fed with bottles during the first 6 months postpartum						
Face-to-face	2 trials	115/259 (44.4%)	155/260 (59.6%)	RR 0.65 (0.34 to 1.23)	209 fewer per 1000 (from 137 more to 393 fewer)	Low
Face-to-face and telephone	3 trials	288/490 (58.8%)	313/461 (67.9%)	RR 0.77 (0.57 to 1.03)	156 fewer per 1000 (from 20 more to 292 fewer)	Low

##### Provider

Sub-group analysis was conducted according to whether the intervention was delivered by lay, non-lay personnel, or by both (Table 5). The largest effect was on reducing the risk of women not initiating breastfeeding within the first hour when counselling was delivered by non-lay personnel, the reduction being 42% based on low quality evidence from two trials (RR 0.58, CI 0.37, 0.90). As can be seen in Table 5, there were smaller effects of non-lay counselling on any breastfeeding at both assessed time-points. Counselling by lay, or combined lay and non-lay personnel reduced the risk of women stopping exclusive breastfeeding at 4 to 6 weeks by about a third, although the quality of evidence was low (lay: RR 0.64, CI 0.42,0.97; lay and non-lay: RR 0.67, CI 0.50,0.90), the effects were not statistically significant for exclusive breastfeeding at 6 months.

**Table 5:** Effects of different providers of interventions

Provider of intervention	Number of trials	No of events		Effect		Certainty
		Intervention	Control	Relative (95% CI)	Absolute (95% CI)	
Number of women who do not initiate breastfeeding within 1 h of birth						
Lay	5 trials	987/1755 (56.2%)	1109/1682 (65.9%)	RR 0.79 (0.56 to 1.11)	138 fewer per 1000 (from 73 more to 290 fewer)	Low
Non-lay	2 trials	51/158 (32.3%)	79/136 (58.1%)	RR 0.58 (0.37 to 0.90)	244 fewer per 1000 (from 58 fewer to 366 fewer)	Moderate
Number of women who stop any breastfeeding before 4–6 weeks postpartum						
Lay	4 trials	157/458 (34.3%)	177/438 (40.4%)	RR 0.82 (0.62 to 1.10)	73 fewer per 1000 (from 40 more to 154 fewer)	Low
Non-lay	24 trials (26 comparisons)	1018/3596 (28.3%)	1111/3468 (32.0%)	RR 0.86 (0.77 to 0.96)	45 fewer per 1000 (from 13 fewer to 74 fewer)	Low
Both lay and non-lay	1 trial	57/168 (33.9%)	69/160 (43.1%)	RR 0.79 (0.60 to 1.04)	91 fewer per 1000 (from 17 more to 173 fewer)	Very low
Number of women who stop any breastfeeding before 6 months postpartum						
Lay	3 trials	123/257 (47.9%)	162/248 (65.3%)	RR 0.71 (0.48 to 1.04)	189 fewer per 1000 (from 26 more to 340 fewer)	Moderate
Non-lay	23 trials (24 comparisons)	2359/4158 (56.7%)	1963/3354 (58.5%)	RR 0.94 (0.89 to 0.99)	35 fewer per 1000 (from 6 fewer to 64 fewer)	Low
Both lay and non-lay	3 trials	717/1141 (62.8%)	316/464 (68.1%)	RR 0.95 (0.88 to 1.02)	34 fewer per 1000 (from 14 more to 82 fewer)	Moderate
Number of women who stop exclusive breastfeeding before 4–6 weeks postpartum						
Lay	8 trials (9 comparisons)	382/930 (41.1%)	690/1011 (68.2%)	RR 0.64 (0.42 to 0.97)	246 fewer per 1000 (from 20 fewer to 396 fewer)	Low
Non-lay	21 trials (24 comparisons)	1851/3105 (59.6%)	1626/2576 (63.1%)	RR 0.91 (0.85 to 0.96)	57 fewer per 1000 (from 25 fewer to 95 fewer)	Low
Both lay and non-lay	1 trial (2 comparisons)	67/240 (27.9%)	50/120 (41.7%)	RR 0.67 (0.50 to 0.90)	137 fewer per 1000 (from 42 fewer to 208 fewer)	Moderate
Number of women who stop exclusive breastfeeding before 6 months postpartum						
Lay	10 trials	1241/2219 (55.9%)	1937/2271 (85.3%)	RR 0.67 (0.30 to 1.51)	281 fewer per 1000 (from 435 more to 597 fewer)	Very low
Non-lay	19 trials 21 comparisons)	2314/2827 (81.9%)	2267/2660 (85.2%)	RR 0.97 (0.94 to 0.99)	26 fewer per 1000 (from 9 fewer to 51 fewer)	Low
Both lay and non-lay	2 trials (3 comparisons)	160/261 (61.3%)	133/140 (95.0%)	RR 0.61 (0.18 to 2.05)	371 fewer per 1000 (from 779 fewer to 997 more)	Very low
Number of infants fed with bottles during the first 6 months postpartum						
Lay	2 trials	115/259 (44.4%)	155/260 (59.6%)	RR 0.65 (0.34 to 1.23)	209 fewer per 1000 (from 137 more to 393 fewer)	Low
Non-lay	3 trials	288/490 (58.8%)	313/461 (67.9%)	RR 0.77 (0.57 to 1.03)	156 fewer per 1000 (from 20 more to 292 fewer)	Moderate

##### Setting

For each outcome in sub-group analyses according to whether the intervention was delivered in an urban, rural or both urban and rural setting, there was low quality evidence of effect for urban settings and a lack of statistically significant findings for rural or combined settings (see Table 6).

**Table 6:** Effects of interventions in different settings

Setting of intervention	Number of trials	No of events		Effect		Certainty
		Intervention	Control	Relative (95% CI)	Absolute (95% CI)	
Number of women who do not initiate breastfeeding within 1 h of birth						
Urban	2 trials	80/300 (26.7%)	117/272 (43.0%)	RR 0.59 (0.39 to 0.91)	176 fewer per 1000 (from 39 fewer to 262 fewer)	Low
Rural	1 trial	378/392 (96.4%)	388/402 (96.5%)	RR 1.00 (0.97 to 1.03)	0 fewer per 1000 (from 29 fewer to 29 more)	Moderate
Urban and rural	2 trials	460/931 (49.4%)	455/854 (53.3%)	RR 0.91 (0.64 to 1.29)	48 fewer per 1000 (from 155 more to 192 fewer)	Very low
Number of women who stop any breastfeeding before 4–6 weeks postpartum						
Urban	21 trials (23 comparisons)	978/3428 (28.5%)	984/3032 (32.5%)	RR 0.85 (0.76 to 0.96)	49 fewer per 1000 (from 13 fewer to 78 fewer)	Low
Rural	1 trial	12/25 (48.0%)	7/25 (28.0%)	RR 1.71 (0.81 to 3.63)	199 more per 1000 (from 53 fewer to 736 more)	Low
Number of women who stop any breastfeeding before 6 months postpartum						
Urban	19 trials (20 comparisons)	1444/2680 (53.9%)	1418/2496 (56.8%)	RR 0.91 (0.84 to 0.98)	51 fewer per 1000 (from 11 fewer to 91 fewer)	Low
Rural	2 trials	57/180 (31.7%)	62/203 (30.5%)	RR 1.03 (0.82 to 1.29)	9 more per 1000 (from 55 fewer to 89 more)	Moderate
Urban and rural	1 trial	81/135 (60.0%)	79/135 (58.5%)	RR 1.03 (0.84 to 1.25)	18 more per 1000 (from 94 fewer to 146 more)	Low
Number of women who stop exclusive breastfeeding before 4–6 weeks postpartum						
Urban	23 trials (28 comparisons)	2031/3561 (57.0%)	1893/2810 (67.4%)	RR 0.81 (0.74 to 0.89)	128 fewer per 1000 (from 74 fewer to 175 fewer)	Low
Rural	1 trial	17/25 (68.0%)	13/25 (52.0%)	RR 1.31 (0.82 to 2.08)	161 more per 1000 (from 94 fewer to 562 more)	Low
Number of women who stop exclusive breastfeeding before 6 months postpartum						
Urban	18 trials (21 comparisons)	1757/2500 (70.3%)	1697/2181 (77.8%)	RR 0.87 (0.81 to 0.93)	101 fewer per 1000 (from 54 fewer to 148 fewer)	Low
Rural	2 trials	284/611 (46.5%)	542/617 (87.8%)	RR 0.55 (0.15 to 1.95)	395 fewer per 1000 (from 747 fewer to 835 more)	Very low
Urban and rural	3 trials	984/1251 (78.7%)	1130/1188 (95.1%)	RR 0.77 (0.47 to 1.24)	219 fewer per 1000 (from 228 more to 504 fewer)	Very low
Number of infants fed with bottles during the first 6 months postpartum						
Urban	3 trials	371/573 (64.7%)	424/568 (74.6%)	RR 0.87 (0.80 to 0.94)	97 fewer per 1000 (from 45 fewer to 149 fewer)	Moderate

#### Sub-group analyses: participant characteristics

##### Parity

We compared pooled effects of interventions provided for primiparous women only with those provided for both multiparous and primiparous women (see Table 7). Mostly, interventions provided for primiparous women specifically did not show statistically significant effects whereas those for primiparous and multiparous women did. For example, there was low quality evidence that the risk of women stopping exclusive breastfeeding at 4 to 6 weeks was reduced by 25% (RR 0.75, CI 0.65, 0.86) in primiparous and multiparous women. However, this effect was not significant in primiparous women. Conversely, interventions provided for primiparous women showed a 33% reduction in the number of infants fed with bottles compared to a non-statistically significant finding for interventions provided for primiparous and multiparous women.

**Table 7:** Effects of interventions on women of different parity

Setting of intervention	Number of trials	No of events		Effect		Certainty
		Intervention	Control	Relative (95% CI)	Absolute (95% CI)	
Number of women who do not initiate breastfeeding within 1 h of birth						
Primiparous	1 trial	16/50 (32.0%)	21/50 (42.0%)	RR 0.76 (0.45 to 1.28)	101 fewer per 1000 (from 118 more to 231 fewer)	Very low
Primiparous and multiparous	5 trials	987/1755 (56.2%)	1109/1682 (65.9%)	RR 0.79 (0.56 to 1.11)	138 fewer per 1000 (from 73 more to 290 fewer)	Low
Number of women who stop any breastfeeding before 4–6 weeks postpartum						
Primiparous	9 trials (10 comparisons)	342/1473 (23.2%)	335/1296 (25.8%)	RR 0.85 (0.67 to 1.08)	39 fewer per 1000 (from 21 more to 85 fewer)	Low
Primiparous and multiparous	19 trials (20 comparisons)	834/2641 (31.6%)	955/2684 (35.6%)	RR 0.87 (0.78 to 0.96)	46 fewer per 1000 (from 14 fewer to 78 fewer)	Low
Number of women who stop any breastfeeding before 6 months postpartum						
Primiparous	6 trials	322/521 (61.8%)	357/525 (68.0%)	RR 0.84 (0.68 to 1.04)	109 fewer per 1000 (from 27 more to 218 fewer)	Low
Primiparous and multiparous	18 trials (20 comparisons)	2694/4781 (56.3%)	1899/3304 (57.5%)	RR 0.94 (0.89 to 0.99)	34 fewer per 1000 (from 6 fewer to 63 fewer)	Low
Number of women who stop exclusive breastfeeding before 4–6 weeks postpartum						
Primiparous	10 trials (11 comparisons)	948/1625 (58.3%)	772/1276 (60.5%)	RR 0.88 (0.77 to 1.00)	73 fewer per 1000 (from 0 fewer to 139 fewer)	Low
Primiparous and multiparous	20 trials (24 comparisons)	1355/2679 (50.6%)	1639/2458 (66.7%)	RR 0.75 (0.65 to 0.86)	167 fewer per 1000 (from 93 fewer to 233 fewer)	Low
Number of women who stop exclusive breastfeeding before 6 months postpartum						
Primiparous	7 trials	425/688 (61.8%)	482/687 (70.2%)	RR 0.85 (0.75 to 0.97)	105 fewer per 1000 (from 21 fewer to 175 fewer)	Moderate
Primiparous and multiparous	23 trials (26 comparisons)	3275/4598 (71.2%)	3838/4364 (87.9%)	RR 0.81 (0.73 to 0.90)	167 fewer per 1000 (from 88 fewer to 237 fewer)	Low
Number of infants fed with bottles during the first 6 months postpartum						
Primiparous	2 trials	59/127 (46.5%)	67/104 (64.4%)	RR 0.67 (0.49 to 0.91)	213 fewer per 1000 (from 58 fewer to 329 fewer)	Moderate
Primiparous and multiparous	2 trials	340/538 (63.2%)	390/532 (73.3%)	RR 0.84 (0.71 to 1.00)	117 fewer per 1000 (from 0 fewer to 213 fewer)	Moderate

We were unable to conduct several prespecified sub-group analyses due either to unclear reporting or lack of data. There was unclear reporting for counselling delivered by a provider with specialist training versus those without such training. Most of the interventions appeared to include some elements of anticipatory approaches, however these were not clearly described and therefore it was not possible to undertake analyses for this sub-category of interventions. There were insufficient data to conduct analyses for the following sub-groups: adolescent women (one study, [53], birth by caesarean section (no studies), multiple pregnancies (one study [59]), mothers planning to return to work (two studies [80, 81]), or mothers with high BMI [74].

## Discussion

This review found that breastfeeding counselling is an effective public health intervention to increase rates of any and exclusive breastfeeding up to 6 months postpartum. Counselling interventions had a significant effect on any and exclusive breastfeeding at the two time points assessed, 4 to 6 weeks and 6 months. Furthermore, counselling appeared to be most effective at maintaining exclusive breastfeeding. In terms of optimal timing and frequency, this review indicates that counselling delivered at least four times in the postnatal period (with or without an antenatal component) is more effective than counselling delivered in the antenatal period only and/or fewer than four times. Face-to face counselling appears to be more effective than telephone counselling. There were mixed findings in terms of who provides the counselling but the largest effects were for lay or combined lay and non-lay providers on exclusive breastfeeding at 4 to 6 weeks postpartum. We also found that counselling interventions appear to be effective in urban settings and when both primiparous and multiparous women are included.

The overall finding of this review that counselling reduced the risk of women stopping any and exclusive breastfeeding is in agreement with other systematic reviews of similar interventions that aim to increase breastfeeding rates [11, 12]. This review differs from others in that it has focused specifically on counselling interventions that are delivered directly to women, and are interactive and support women with their decision-making, rather than including studies of interventions that only provided education [11] and/or systems level interventions, such as implementation of WHO/UNICEF Baby friendly initiative [14, 15, 82]. As others have suggested [12, 83], it is crucial to identify the elements of these important but heterogeneous interventions that are effective and this review contributes specifically to this.

The effect size found in this review is smaller than that found in others [11, 14] which may be partly due to the focus on one type of intervention, as previous reviews have found multicomponent interventions to be most effective [14, 15, 82]. It is also likely that counselling interventions to encourage and support breastfeeding are affected by the context within which breastfeeding occurs. In particular, the interactive and responsive nature of counselling may mean it is more effective in areas where breastfeeding is seen as the norm and when women are already motivated to breastfeed. One implication of this is that breastfeeding initiation needs to be considered alongside initiatives to improve breastfeeding duration and exclusivity.

Furthermore, this review included relatively few studies from low- and middle-income countries where breastfeeding rates are generally higher (approximately a third of studies and a quarter of participants). In countries where breastfeeding rates are high interventions to promote and support breastfeeding appear to have greater effect, particularly in maintaining exclusive breastfeeding [11, 12, 82]. This may partially explain both the smaller effect size and the greater effect of counselling interventions on exclusive rather than on any breastfeeding found in this review. The existing provision of healthcare in each country or area may also be relevant. In a review including only interventions delivered in low- and middle-income countries, Olufunlayo et al. [82] estimate a much greater effect, a two to three-fold increase in exclusive breastfeeding at 6 months, and as they suggest, there may be more potential for interventions to be effective where standard healthcare provision is lacking.

Counselling emphasises interactions with individual mothers to support their decision-making, which means that the content and style of the intervention inevitably varies for different women at different times. Whilst at the micro level this is variable, at the macro level it is possible to identify criteria that make it effective. The subgroup analyses in this review found that counselling was most effective when delivered face-to-face and was not effective when only delivered antenatally. These findings are perhaps not surprising given the relational nature of counselling. It is highly likely that relations between health workers and mothers can be established more easily face-to-face, and when provided across the childbearing continuum, may develop in a way that enhances women’s confidence and self-efficacy. This needs careful consideration as in many countries the reduced healthcare provision for breastfeeding mothers may mean many only receive breastfeeding counselling before birth, and telephone support may be considered a cheaper and easier option.

More frequent counselling may also enable women and healthcare workers to build rapport and may be important in enabling counsellors to respond in a timely way when women encounter challenges, particularly in the early days after birth. This review found that when counselling was provided four or more times it was more effective for maintaining exclusive breastfeeding (≥ 4 times 31% compared to < 4 times 8%). However, there was huge variation in the frequency of counselling (from 1 to 24 contacts) and a counselling intervention that is only delivered once in the hospital setting is very different to one provided regularly within an established relationship. This is similar to the recent review of support interventions, which found greatest effect on exclusive breastfeeding when the intervention was provided between four and eight times [12]. While an optimal number of counselling sessions could not be definitely pinpointed from the evidence, the World Health Organization guideline recommends counselling is provided at least six times and more if needed [22] based on the available evidence and other considerations. It is also likely that there is interplay between frequency of counselling and its timing to coincide with the period when women encounter most challenges, such as in the early period after birth when they are developing breastfeeding skills and/or at times when they may be considering introducing other foods or returning to work [84] .

### Strengths and limitations

This systematic review was conducted robustly, included a large number of studies and was limited to randomised controlled trials to ensure it is based on the strongest evidence available. However, findings should be treated with caution as the evidence was mostly of low quality due to high or unclear risk of bias of the included trials. This is partly attributable to a lack of blinding which would not be feasible with such an intervention. Breastfeeding counselling is often part of complex multicomponent interventions and, as in most systematic reviews of breastfeeding interventions [12–14], there was considerable heterogeneity of the counselling interventions within the review. This included frequency and timing of counselling, who delivered the intervention (e.g., a qualified health professional or a lay person), and if they were specifically trained in breastfeeding counselling. Additionally, reporting of interventions within studies was not always comprehensive, clear or sufficiently detailed, making it difficult to identify the components of the counselling intervention and/or care received by control groups. In particular training of non-lay providers in breastfeeding counselling knowledge and skills was poorly described which meant we could not include this aspect in a meta-analysis. We could not conduct planned sub-group analysis by mode of birth because there were no studies of the effectiveness of breastfeeding counselling following caesarean birth. We also found no studies of breastfeeding counselling that measured breastfeeding outcomes at 24 months.

## Conclusions

The findings of this systematic review demonstrate that counselling interventions are effective for improving breastfeeding practices with the greatest effect on exclusive breastfeeding. The review has informed a recent global guideline [22]. Recommendations include that breastfeeding counselling should be provided face-to-face, and, in addition, may be provided by telephone, both antenatally and postnatally to all pregnant women and breastfeeding mothers. However, in order to inform scale-up and sustainability globally, there is a need to further understand the elements of interventions such as counselling and their effectiveness in different contexts and circumstances.

## Supplementary information

**Additional file 1.** Search strategy.

**Additional file 2.** Characteristics of included studies and description of intervention and comparison.

## Data Availability

All data generated or analysed during this study are included in this published article and its Additional information files.

## Abbreviations

CI: Confidence interval
ICC: Intra-cluster correlation coefficient
RR: Relative risk
WHO: World Health Organization
