# Supplementary material for: Counselling interventions to enable women to initiate and continue breastfeeding: a systematic review and meta-analysis
Source: Int Breastfeed J. 2019 Oct 21;14:42. doi: 10.1186/s13006-019-0235-8 (PMC6805348; doi:10.1186/s13006-019-0235-8)
Supplement: Supplementary file 1 — Additional file 1. Search strategy. [file 13006_2019_235_MOESM1_ESM.docx]

**Additional file 1: search strategy**

**MEDLINE**

Searched using Ovid on 05/02/2018

1. exp Breast Feeding/

2. breastfeed*.mp. [mp=title, abstract, original title, name of substance word, subject heading word, keyword heading word, protocol supplementary concept word, rare disease supplementary concept word, unique identifier, synonyms]

3. breastfed.mp. [mp=title, abstract, original title, name of substance word, subject heading word, keyword heading word, protocol supplementary concept word, rare disease supplementary concept word, unique identifier, synonyms]

4. breast-feed*.mp. [mp=title, abstract, original title, name of substance word, subject heading word, keyword heading word, protocol supplementary concept word, rare disease supplementary concept word, unique identifier, synonyms]

5. breast-fed.mp. [mp=title, abstract, original title, name of substance word, subject heading word, keyword heading word, protocol supplementary concept word, rare disease supplementary concept word, unique identifier, synonyms]

6. breast feed*.mp. [mp=title, abstract, original title, name of substance word, subject heading word, keyword heading word, protocol supplementary concept word, rare disease supplementary concept word, unique identifier, synonyms]

7. breast fed.mp. [mp=title, abstract, original title, name of substance word, subject heading word, keyword heading word, protocol supplementary concept word, rare disease supplementary concept word, unique identifier, synonyms]

8. infant feed*.mp. [mp=title, abstract, original title, name of substance word, subject heading word, keyword heading word, protocol supplementary concept word, rare disease supplementary concept word, unique identifier, synonyms]

9. exp MILK, HUMAN/

10. LACTATION/

11. lactat*.mp. [mp=title, abstract, original title, name of substance word, subject heading word, keyword heading word, protocol supplementary concept word, rare disease supplementary concept word, unique identifier, synonyms]

12. exp Counseling/

13. counsel*.mp. [mp=title, abstract, original title, name of substance word, subject heading word, keyword heading word, protocol supplementary concept word, rare disease supplementary concept word, unique identifier, synonyms]

14. support.ab.

15. Social Support/

16. anticipatory guidance.mp.

17. exp Directive Counseling/

18. Health Promotion/

19. Health Education/

20. randomized controlled trial.pt.

21. controlled clinical trial.pt.

22. randomized.ab.

23. placebo.ab.

24. drug therapy.fs.

25. randomly.ab.

26. trial.ab.

27. groups.ab.

28. 1 or 2 or 3 or 4 or 5 or 6 or 7 or 8 or 9 or 10 or 11

29. 12 or 13 or 14 or 15 or 16 or 17 or 18 or 19

30. exp animals/ not humans.sh.

31. 20 or 21 or 22 or 23 or 24 or 25 or 26 or 27

32. 31 not 30

33. 28 and 29 and 32

**EMBASE**

Searched using Ovid on 5/2/18

1. exp breast feeding/

2. exp breast feeding education/

3. breastfeed*.mp. [mp=title, abstract, heading word, drug trade name, original title, device manufacturer, drug manufacturer, device trade name, keyword, floating subheading word]

4. breastfed.mp. [mp=title, abstract, heading word, drug trade name, original title, device manufacturer, drug manufacturer, device trade name, keyword, floating subheading word]

5. breast-feed*.mp. [mp=title, abstract, heading word, drug trade name, original title, device manufacturer, drug manufacturer, device trade name, keyword, floating subheading word]

6. breast-fed.mp. [mp=title, abstract, heading word, drug trade name, original title, device manufacturer, drug manufacturer, device trade name, keyword, floating subheading word]

7. breast feed*.mp. [mp=title, abstract, heading word, drug trade name, original title, device manufacturer, drug manufacturer, device trade name, keyword, floating subheading word]

8. breast fed.mp. [mp=title, abstract, heading word, drug trade name, original title, device manufacturer, drug manufacturer, device trade name, keyword, floating subheading word]

9. exp infant feeding/

10. infant feed*.mp. [mp=title, abstract, heading word, drug trade name, original title, device manufacturer, drug manufacturer, device trade name, keyword, floating subheading word]

11. exp lactation/

12. lactat*.mp. [mp=title, abstract, heading word, drug trade name, original title, device manufacturer, drug manufacturer, device trade name, keyword, floating subheading word]

13. exp breast milk/

14. exp counseling/

15. counsel*.mp. [mp=title, abstract, heading word, drug trade name, original title, device manufacturer, drug manufacturer, device trade name, keyword, floating subheading word]

16. social support/

17. support.ab.

18. exp anticipatory guidance/

19. anticipatory guidance.mp. [mp=title, abstract, heading word, drug trade name, original title, device manufacturer, drug manufacturer, device trade name, keyword, floating subheading word]

20. exp directive counseling/

21. health promotion/

22. health education/

23. CROSSOVER PROCEDURE/

24. DOUBLE BLIND PROCEDURE/

25. SINGLE BLIND PROCEDURE/

26. RANDOMIZED CONTROLLED TRIAL/

27. crossover$.ti,ab.

28. (cross adj over$).ti,ab.

29. placebo$.ti,ab.

30. (doubl$ adj blind$).ti,ab.

31. allocat$.ti,ab.

32. random$.ti,ab.

33. trial$.ti.

34. 1 or 2 or 3 or 4 or 5 or 6 or 7 or 8 or 9 or 10 or 11 or 12 or 13

35. 14 or 15 or 16 or 17 or 18 or 19 or 20 or 21 or 22

36. 23 or 24 or 25 or 26 or 27 or 28 or 29 or 30 or 31 or 32 or 33

37. 34 and 35 and 36

**CINAHL**

Searched using EBSCO on 05/02/18

| S42 | S13 AND S24 lacAND S41 |
| --- | --- |
| S41 | S25 OR S26 OR S27 OR S28 OR S29 OR S30 OR S31 OR S32 OR S33 OR S34 OR S35 OR S36 OR S37 OR S38 OR S39 OR S40 |
| S40 | (MH "Quantitative Studies") |
| S39 | AB (allocat* N1 random*) |
| S38 | (MH "Placebos") |
| S37 | AB (placebo) |
| S36 | (MH "Random Assignment") |
| S35 | AB(random* N1 allocat*) |
| S34 | AB(random* N1 control* N1 trial*) |
| S33 | AB(singl* N1 blind*) |
| S32 | AB(doubl* N1 mask*) |
| S31 | AB(doubl* N1 blind*) |
| S30 | AB(tripl* N1 mask*) |
| S29 | AB(tripl* N1 blind*) |
| S28 | AB(trebl* N1 mask*) |
| S27 | AB(clinic* N1 trial*) |
| S26 | (MH "Clinical Trials+") |
| S25 | exp CLINICAL TRIALS/ |
| S24 | S14 OR S15 OR S16 OR S17 OR S18 OR S19 OR S20 OR S21 OR S22 OR S23 |
| S23 | (MH "Peer Counseling") |
| S22 | (MH "Health Education") |
| S21 | (MH "Health Promotion") |
| S20 | directive counseling |
| S19 | AB (Anticipatory Guidance) |
| S18 | (MH "Anticipatory Guidance") |
| S17 | AB (support) |
| S16 | (MH "Support, Psychosocial") |
| S15 | counsel* |
| S14 | (MH "Counseling") |
| S13 | S1 OR S2 OR S3 OR S4 OR S5 OR S6 OR S7 OR S8 OR S9 OR S10 OR S11 OR S12 |
| S12 | (MH "Milk, Human+") |
| S11 | lactat* |
| S10 | (MH "Lactation") |
| S9 | infant feed* |
| S8 | (MH "Infant Feeding+") |
| S7 | breast fed |
| S6 | breast feed* |
| S5 | breast-fed |
| S4 | breast-feed* |
| S3 | breastfed |
| S2 | breastfeed* |
| S1 | (MH "Breast Feeding+") |

**CENTRAL**

Searched on 05/2/18

#1 MeSH descriptor: [Breast Feeding] explode all trees 1724

#2 breastfeed* or breastfed or breast feed* or breast fed or breast-feed* or breast-fed 6293

#3 infant feed* 5719

#4 MeSH descriptor: [Lactation] explode all trees 556

#5 MeSH descriptor: [Milk, Human] explode all trees 920

#6 MeSH descriptor: [Counseling] explode all trees 4907

#7 (counsel* or support*) ti,ab 3358

#8 anticipatory guidance 122

#9 MeSH descriptor: [Directive Counseling] explode all trees 961

#10 MeSH descriptor: [Health Promotion] explode all trees 3086

#11 MeSH descriptor: [Health Education] explode all trees 16310

#12 #1 or #2 or #3 or #4 or #5 in Trials 7165

#13 #6 or #7 or #8 or #9 or #10 or #11 in Trials 18131

#14 #12 and #13 in Trials 459

**WHO ICTRP**

Searched on 5/2/18

(breastfeed* or breast-feed* OR breast feed* OR lactat* OR breast fed)
AND (support* OR counsel* OR guid*)

**Clinicaltrials.gov**

Searched on 5/2/18

Condition: Breastfeeding OR breast-feed OR breast feed OR lactat OR infant feed

AND

Intervention: counsel OR support OR guidance
