# Supplementary material for: Counselling interventions to enable women to initiate and continue breastfeeding: a systematic review and meta-analysis
Source: Int Breastfeed J. 2019 Oct 21;14:42. doi: 10.1186/s13006-019-0235-8 (PMC6805348; doi:10.1186/s13006-019-0235-8)
Supplement: Supplementary file 2 — Additional file 2. Characteristics of included studies and description of intervention and comparison. [file 13006_2019_235_MOESM2_ESM.docx]

**Additional file table 2: characteristics of included studies and description of intervention and comparison**

| **Author**  **Year published**  **Country** | **Study design**  **Year of study** | **Participants**  Number randomised intervention/ control | **Mode, frequency, (length of sessions) and who provided the intervention** | **Description of intervention** | **Description of comparison** |
| --- | --- | --- | --- | --- | --- |
| Agrasada  2005 [1]  Philippines | Parallel RCT  3 arms (2 relevant for this review)  2001- 02 | Low birth weight babies  68/69 | One-to-one, face-to-face by trained peer counsellor postnatal on days: 3–5, 7–10, 21, and at 1.5 months, and monthly up to 5.5 months. | Counselling using semi-structured guide, information on benefits of exclusively breastfeeding to 6 months. Assist with managing BF problems. | No counselling (no additional information provided). |
| Ahmadi  2016 [2]  Iran | Parallel RCT  2 arms  2014 - 15 | Preterm babies  62/62 | Face-to-face by researcher, PN from start of oral feeding 5 times (30 mins) on consecutive days. | Counselling included, greet and tell clients about their choices, help clients choose. Used model, pamphlets and incentives package. Included information on benefits of EBF, BF techniques, prevention of lactation problems. | Conventional training from ward staff. |
| Aidam  2005 [3]  Ghana | Parallel RCT  3 arms  2002 | AN & PN  74/85 | Face-to-face by 2 nurses and a nutritionist who had successfully BF. 2 AN in groups of 2-4 women (20 mins) and 9 PN at 1, 2, 4, 6, 8, 12, 16, 20, and 24 weeks. | Information from WHO/UNICEF BF counselling training manual on benefits of EBF (dangers of pre-lacteal feeds, adequacy of breastmilk for 6 months), early BF initiation, importance of on-demand and frequent feeds, BF techniques and prevention and management of lactation problems. Additional topics addressed as raised by mothers. | Non-BF educational support - directed to welfare clinic for BF support. Received 2AN and 9PN visits. |
|  |  | PN  72/85 | 9 PN at 1, 2, 4, 6, 8, 12, 16, 20, and 24 weeks. | Same as above but no BF counselling in AN sessions |  |
| Aksu  2011 [4]  Turkey | Parallel RCT  2 arms  2008 | 33/33 | One extra session (30 mins) delivered by two trained lay supporters face-to-face on day 3 at home. | The training topics were the same topics that were covered in the standard provision. Based on the WHO/UNICEF breastfeeding counselling/lactation management courses - information -production of breastmilk, benefits of EBF, BF techniques, adequacy of BF for 6 months, breastmilk substitutes, problems and management of these. | Standard care included one session (20-30mins) soon after birth based on the WHO/UNICEF breastfeeding counselling/lactation management courses. |
| Anderson 2005 [5]  USA | Parallel RCT  2 arms  2003 - 04 | Low income Latina community  90/92 | One-to-one, face-to-face by trained peer counsellors (plus telephone if needed). AN: 1st within week of assignment; 2^nd^ before 36 weeks, 3^rd^ during 36th week. PN: at least once a day in hospital, plus 9 visits (3 in 1st week, 2 in 2nd week, 1 per week from 3-6wks). | AN benefits of EBF, avoiding bottles/pacifiers, early initiation and successful BF, why EBF babies do not need water during the first 6 months of life, infant cues for readiness to breastfeed, BF techniques. PN hands-on breastfeeding support and counselling according to the mother’s needs. Involved family. | Routine BF education and support in a hospital certified as WHO/UNICEF Baby Friendly – includes technical BF and support with problems. Mothers can also telephone 24 hrs a day for support and counselling from a staff nurse/lactation consultant. |
| Ara 2017 [6]  Bangladesh | Cluster RCT  2014 - 16 | Living in urban slums  192/186 | At least 10 scheduled visits (20-40 mins). Group with family members, face-to-face by lay supporters with training. 3 AN, PN 1^st^ within 48hrs of birth, 2^nd^ 10-14 days later, 3^rd^ 24-28 days later, 4 visits in 2nd to 6th months. Extra as required. | AN: early contact, initiation of BF, discouraged pre-lacteal feeds, overcoming BF problems. Fortnightly group meetings: encouraged care, proper nutrition, to attend antenatal care, colostrum feeding and EBF. Frequent home visits to support mothers with EBF. | No peer counselling (no further details provided). |
| Bonuck 2013 [7]  BINGO  USA | Parallel RCT  4 arms  (LC, EP both)  2008-11 | Low income  666 randomised in 1:3:3:1 ratio (n for each arm not available) | One-to-one face-to-face 2 AN and 1 PN hospital visit, regular phone calls to 3 months by lactation consultant. | AN: rapport building and education, practical aspects of BF electronically prompted guidance. PN: 1-week routine paediatric visit and optional home visits. | Usual care – no explicit BF promotion or support. Lactation consultant available weekdays to support women with BF difficulties. |
| Bonuck  2006 [8]  MILK  USA | Parallel RCT  2 arms  2000 - 02 | 188/194 | One-to-one, face-to-face 2 AN and 1 PN hospital and/or home visit (60 mins), telephone support for up to 12 months by lactation consultants. | AN: rapport and educational - feeding intentions and benefits of BF, initiation of BF. PN: practical help, avoiding BF complications. Confidence, adequate intake. Later contacts - fatigue, feeding in public, returning to school or work. Sources of support e.g. families, schools, workplaces. | Usual care (no contact with study lactation consultants) |
| Bonuck 2014 [7]  PAIRINGS  USA | Parallel RCT  2 arms  2008-11 | 137/138 | One-to-one, 2 AN and 1 PN hospital visit, regular phone calls up to 3 months by lactation consultant. | AN: rapport building and education, practical aspects of breastfeeding.  PN: 1-week routine paediatric visit and optional home visits. | Usual care – no explicit BF promotion or support. Lactation consultant available weekdays to support women with BF difficulties. |
| Brent  1995 [9]  USA | Parallel RCT  2 arms  Year of study not provided. | Low income  58/57 | One-to-one, face-to-face, 2-4 AN (10-15mins), PN: daily as inpatient by a lactation consultant. Telephone at 48hrs after discharge, visit to clinic at 1 week, each health supervision visit until weaning or 1 year. | Responded to mother’s needs e.g. BF practical management, early initiation, demand feeding etc. avoidance of artificial teats. | Standard care at the hospital – optional AN BF classes. PN BF instruction by nurses and physicians. |
| Cangol 2017 [10]  Turkey | Parallel RCT  2 arms  2014 | 50/50 | Face-to-face, 1 group AN (20 mins) between 32-36wks and 3 one-to-one PN (20mins) 1st PN day, 4-6 PN week and telephone contact in 4th month by researcher trained by motivational interviewing expert. | BF motivational programme using motivational interviewing to illicit behavioural change. Helps mother to understand own problems and take action to change and believe that change is possible | Trained in breast self-examination |
| Carlsen 2013 [11]  Denmark | Parallel RCT  2 arms  2010-12 | 108/118 | One-to-one telephone by a certified lactation consultant. PN 1st (20 mins) wk1, then 3 in 1st month (5-10 mins), then every 2nd week, minimum of 9. | All contacts followed a structured design posing questions of physical and psychological aspects related to breastfeeding and the well-being of the mother and child. Difficulties discussed and solutions identified. | Standard BF support. Included contact with a health visitor or a midwife within 1^st^ week after birth. Home visits during the first 18 months to examine wellbeing of baby and support breastfeeding. |
| Chan  2016 [12]  Hong Kong | Parallel RCT  2 arms  Year of study not provided. | 35/36 | Group AN Self-efficacy-based BF educational programme and one-to-one PN telephone counselling (30–60 mins) was provided to the participants 2wks by researcher. | Self-efficacy-based BF educational programme focusing on evaluating their emotional/physiological condition and BF status. Addressed problems, such as fear and pain, with the aim of correcting misconceptions. Coping strategies were reinforced and emotional support provided. Positioning, infant cues of hunger, and frequency of BF. Advice was given and BF practices were encouraged. | Usual care - BF support provided by MWs in hospital, seeking help from lactation consultant, PN follow up by midwives and doctors. |
| Chapman  2004 [13]  USA | Parallel RCT  2 arm  2000-02 | Low income Latina mostly Puerto Rican origin  113/106 | One-to-one, face-to-face 3 AN, daily in hospital, up to 11 PN - 3 in 1st week, 2 in 2nd, 3rd and 4th weeks, weekly 5th and 6th week, telephone contact 2-3 months and extra visits as needed by peer counsellors. | AN: benefits of BF, discussion of common BF myths. BF video. PN: in hospital hands-on assistance with practical aspects of BF, infant feeding cues, expected feeding frequency, signs of adequate BF, problem solving. | WHO/UNICEF Baby Friendly hospital. Individualized BF information in response to questions, practical assistance and education from nurses, written BF educational materials, and access to an IBCLC for serious BF problems. Could telephone nurse to ask BF questions. |
| Chapman 2013 [14]  USA | Parallel RCT  2 arm  2006-09 | Women with high BMI  103/103 | One-to-one, face-to-face 3 AN, daily in hospital, up to 11 PN home visits by peer counsellors plus telephone calls if needed. | AN: assessment of previous BF experience, personalised BF education, risks of formula feeding,  PN: in hospital | WHO/UNICEF Baby Friendly hospital. Usual care - AN brief BF discussions during routine clinical appointments and written materials. Routine perinatal care from staff nurses and lactation consultants available as needed. |
| Ciftci  2012 [15]  Turkey | Parallel RCT  2 arms  2008-09 | Working mothers  34/33 | One-to-one, face-to-face, 1 training session (60 mins) 2 weeks before mothers started work (after 2 ½ months) appears to be by researcher (not clear). | Training programme advantages of breastmilk and BF, benefits to mother and infant, techniques for expressing and storing breastmilk and a training booklet. | Home visits were carried out for data collection but did not receive the training programme. |
| Coutinho  2005 [16]  Brazil | Parallel RCT  2 arms  2001 | 175/175 | One-to-one, face-to-face 10 visits on days 3, 5, 7, 15 and 30 (30 mins) and every 2 weeks during 2nd month, once a month 3-6 months by trained home visitors | Encourage EBF for 6 months and continued BF for at least 2 years, to answer questions, discuss doubts, use the booklet for the basis of discussions of key topics relevant to the infants age. Observe positioning, flow of milk and baby's satisfaction. Refer if there were difficulties they could not resolve. Involve family members in practical support. | WHO/UNICEF trained health professionals and support staff. Hospital based staff to encourage EBF for 6 months and continued BF for at least 2 years, skin to skin, practical support, answer questions, show video daily in hospital advice to return if difficulties. |
| Davies-Adetugbo 1997 [17]  Nigeria | Parallel RCT  2 arms  1995-96 | Infants with diarrhoea  84/85 | One-to-one, face-to-face 3PN/1st (20mins), 2nd and 3rd (30 mins) by community health workers and researchers. | Lactation problems discussed and solved. BF counselling importance of EBF and that infants can survive well on breastmilk only for 6 months. Demonstrations and assistance for positioning and attachment of baby to breast, and hand expression. | Advice on diarrhoea including continuation of breastfeeding, avoidance of use of feeding bottles, breastmilk only. |
| Dennis  2002 [18]  Canada | Parallel RCT  2 arms  1997-98 | 132/126 | One-to-one, telephone within 48hrs after hospital discharge and as necessary after that by trained lay supporter. | Paired with peer volunteer from an existing organisation - contact depending on mother's needs. | Usual care, hospital and community PN services provided by nursing and medical staff, hospital clinic managed by a lactation consultant, telephone BF support line managed by hospital nursing staff and support by public health nurses, community physicians and pediatricians. |
| De Oliveira 2006 [19]  Brazil | Parallel RCT  2 arms  2003 | 74/137 | One-to-one, face-to-face & group (no more than 2 mother-infant pairs)  1PN (30 mins) by nurse or lactation consultant | BF technique using WHO breastfeeding counselling principles – positioning and attachment, milk expression. Pictures, dolls, and a model breast used for demonstration. Mothers were encouraged to breastfeed their infants during the intervention. | WHO/UNICEF Baby Friendly hospital. Usual care – assistance with first feed, BF technique, practical assistance and women with difficulties assisted by lactation consultants. |
| Di Napoli  2004 [20]  Italy | Parallel RCT  2 arms  2000-01 | 303/302 | One-to-one, face-to-face home visit within 7 days of discharge (30 mins) and telephone counselling by midwives with WHO/UNICEF training | No details provided. | No specific interventions provided. |
| Edwards 2013 [21]  USA | Parallel RCT  2 arms 2001-04 | Low income African American  124/124 | One-to-one, face-to-face AN, perinatal (present during labour) and PN home visits to 3 months by doula with training | Focused on multiple aspects of maternal and infant well-being with breastfeeding advocacy and support as primary. Benefits of BF Present during labour, delivery, and after birth to encourage 1^st^ BF | Usual care – not described. |
| Elliott-Rudder  2014 [22]  Australia | Cluster  Parallel RCT  2 arms  2008-09 | 154/176 | One-to-one, face-to-face with each BF mother who attended a general practice intervention site for their infant to be immunised at 2, 4 or 6 months by practice nurses with WHO/UNICEF. | Structured conversation to support continuation of BF using a Conversation Tool flowchart that used a motivational interviewing approach. Informed of the recommendation for EBF to 6 months and maintenance to 1 to 2 years and asked ‘How would that work for you?’ According to the mother’s response provided targeted proactive conversational action. | Maternity hospital not Baby Friendly accredited but each hospital had a International Board Certified Lactation Consultant and registered midwives encouraged mothers to BF. |
| Fu 2014 [23]  Hong Kong | Cluster  Parallel RCT  3 arms  2010-11 | Hospital  191/264 | One-to-one, face-to-face 3PN (30-45 mins) 2 in 24hrs & 1 in next 24hrs prior to discharge by trained researchers (either midwives or lactation consultants). | Information - benefits of EBF, physiology and common BF problems. BF techniques, e.g. P&A, latching and attachment, assessing feeding behaviours, expression. Encouraged to ask questions and raise concerns. | Usual care - group postnatal lactation education provided by a midwife or lactation consultant, assistance with BF problems, post-discharge follow- up, either at the outpatient clinic or at the nearest Maternal and Child Health Centre. Information on available peer-support groups is also provided upon hospital discharge. |
|  |  | Telephone  269/264 | One-to-one, telephone PN (20-30 mins) within 72hrs of discharge and then for 4 weeks by trained researchers (either midwives or lactation consultants). | Early support sessions - general BF knowledge, infant feeding patterns, the physical and emotional health, managing problems. Later sessions – feeding in public, expressing and return to work. EBF encouraged. |  |
| Gijsbers  2006 [24]  Netherlands | Cluster  Parallel RCT  2 arms  2002 - 03 | Relative with asthma  58/55 | One-to-one, face-to-face 2AN 3^rd^, 6^th^ and 8^th^ month and 1PN within 4 weeks (60 mins) by trained researcher. | Motivated women to BF and to postpone solids for at least 6 months. Used booklet to review - based on principles of Attitude-Social influence-self efficacy model (ACE model) and focus groups from previous study. | Usual care - The guidelines of the Dutch College of General Practitioners recommend breastfeeding for six months for all babies in the Netherlands. Visited once AN no information about BF. |
| Graffy 2004 [25]  UK | Parallel RCT  2 arms  1995-98 | 363/357 | One-to-one, face-to-face 1AN/ PN telephone or visit if requested (30-45 mins) trained lay women who had BF. | AN visit gave contact card and 2 x leaflets (NCT and HEA) - non directive approach, strengthening mothers' confidence. PN support offered by telephone or home visit | Usual care not described |
| Grossman  1990 [26]  USA | Parallel quasi  RCT  2 arms  1986-87 | Low income  49/48 | One-to-one, face-to-face in hospital and telephone PN days 2, 4, 7, 10 days and 3 weeks plus telephone help-line by a registered nurse or paediatrician. | Educational booklet given after birth. Questions were directed towards BF difficulties known to occur commonly. Support for those with problems from a lactation clinic. | Routine postnatal teaching on infant care and feeding by obstetric nursing staff |
| Haider 2000 [27]  Bangladesh | Cluster  Parallel RCT  2 arms  1996 | 363/363 | One-to-one, face-to-face 2AN in last trimester 15PN x 4 in 1^st^ month, 2 weekly months 2-5 (20-40 mins) by peer counsellors with experience of BF and training. | Benefits of EBF for 5 months. Nutrition during pregnancy to support enhanced lactation. Early initiation of breastfeeding and discouraging prelacteal and post lacteal foods. Addressed the mother’s specific needs or difficulties at that time, provided continued support for EBF and could refer to BF supervisors. | No description provided. |
| Jones  1986 [28]  UK | Parallel RCT  2 arms  Year of study not provided. | 228/355 | One-to-one, face-to-face Early weeks - up to 4 weeks by health professional – lactation nurse. | Assist and encourage mothers to initiate and continue BF. Technical help to BF early, attempt to prevent complications. Advice expertise, general support and encouragement. | Usual care – not described |
| Khresheh  2011 [29]  Jordan | Parallel RCT  2 arms  2008-09 | 72/68 | One-to-one, 3PN - face-to-face 2 hrs after birth (60 mins), telephone at 2 & 4 months by researcher | Verbal information, discussion and questions, practical demonstration of BF techniques, education on benefits of BF, importance of EBF, positioning and attachment, common BF problems and infant growth and development. | Routine postnatal care – not described. |
| Kimani-Murage 2017 [30]  Kenya | Cluster  Parallel RCT  2 arms  2012-14 | Living in urban slums  771/784 | One-to-one, face-to-face 7AN monthly to 36wks then weekly. 17PN weekly for 1 month then monthly to 1 year except biweekly in 5th month by community Health Workers with training. | Counselling was also informed by the stages of change model. Personalized - maternal nutrition, initiation of BF, positioning and attachment, EBF, frequency and duration of BF, expressing, storage, handling and feeding of expressed breastmilk, age-appropriate complementary feeding. | Standard care – counselling from Community health Workers on primary health care, AN and PN |
| Kronborg 2007 [31]  Denmark | Parallel RCT  2 arms  2004 | 780/815 | One-to-one, face-to-face 1 to 3PN within 5 weeks. 1 soon after coming home from hospital, to assess need for support in later visits by health professional with training. | Address mother's concerns - BF technique and learning to know the baby, BF and interpretation of baby’s cues, sufficient milk and interaction with the baby. Booklet used | Usual practice consisting of one or more non-standardized visits. Health visitors were informed about the project but did not take part in the training |
| Kupratakul 2010 [32]  Thailand | Parallel RCT  2 arms  2009 | 40/40 | Face-to-face 1AN group (3 hr) and 8PN one-to-one telephone 7 and 14 days, 1, 2, 3, 4, 5, and 6 months by researcher. | Knowledge sharing with empowerment on AN education and PN support strategies – four steps, discovering reality, critical reflection, taking charge, and holding on to discover their own success in managing themselves to BF and learn about others experiences. | Routine BF education and nipple assessment, benefits of BF. |
| Lynch 1986 [33]  Canada | Parallel RCT  2 arms  1984 | 135/135 | One-to-one, face-to-face PN 1st visit within 5 days of discharge, then weekly telephone calls in 1st month, then monthly to 6 months and followed up with home visits and additional calls when necessary by lactation consultant. | Condition of breasts, baby’s feeding and problems. Observed BF, positioning, latching, nipple care and expressing and provided lifestyle advice and advice for problems (e.g. engorgement, mastitis). Mothers could telephone the service if problems developed. | Standard care - AN classes, routine visit by a public health nurse after hospital discharge to discuss problems BF and advice about ways to increase milk supply as determined by the mother’s concerns and questions. |
| Mattar  2007 [34]  Singapore | Parallel RCT  3 arms ( 2 relevant for this review)  2002-04 | 123/146 | One-to-one, face-to-face AN (15 mins + video) by lactation counsellor (no details). | Booklet, video and counselling - benefits of BF, practical advice re BF, expressing and management of common problems. Counselling - 'examined nipples to assess adequacy and answered questions on breastfeeding'. | Standard care including access to PN BF support, no booklet, no video, no counselling. |
| McDonald 2010 [35]  Australia | Parallel RCT  2 arms  2000-01 | 425/424 | One-to-one, face-to-face 6PN 1st session in hospital, then weekly home visit and twice weekly telephone calls until 6 weeks by research midwife. | Educational session in hospital with video - positioning and attachment, common BF problems, growth and development, crying patterns and settling techniques. Unstructured telephone calls and home visits to provide informed source of BF support, identify barriers to BF, problems, social support and referral if needed. Encouraged to access local community support services. | Standard care – working towards WHO/UNICEF BHFI accreditation. One or more domiciliary visits by a hospital-based midwife after discharge and before the baby was seven days old, access to outpatient lactation clinics. BF promotional literature and access to videos on establishing BF. |
| McLachlan 2016 [36]  Australia | Cluster  Parallel RCT  3 arms  2012-13 | Home  3335/3449 | One-to-one, face-to-face, early PN within 7 days of hospital discharge by maternal and child health nurses. | Proactive, early, home-based BF support to women identified at risk of breastfeeding cessation. Aim to fill gap between hospital based care and start of maternal and child health care. To build women’s confidence to BF, develop a feeding plan and share contact details in response to women’s needs. | Standard care - 48hrs in hospital, MW visit 1-2 days after discharge - general focus on well-being of mother an infant. Home visit 10 days to 2 weeks after birth with BF assessment, support |
|  |  | Drop-in  2891/3449 | One-to-one, face-to-face, early PN within 7 days of hospital discharge by maternal and child health nurses plus access to drop-in centre. | As above with an additional component—access to a community-based breastfeeding drop-in centre. The drop-in centres were welcoming spaces offering privacy, where women could discuss BF concerns, with the opportunity to meet and learn from other mothers. |  |
| McQueen 2011 [37]  Canada | Parallel RCT  2 arms  2008 | 68/81 | 3PN one-to-one, face-to-face in hospital - 1^st^ within 24 hours, 2^nd^ within 24 hours of 1^st^,  3^rd^ by telephone within 1 week of discharge by researcher (nurse). | Individualised, self-efficacy enhancing sessions delivered in a standardised format (based on Bandura’s social learning theory and Dennis’s BF self-efficacy framework). Intervention tailored to meet mother’s needs. Observation of BF at one of the two in hospital sessions. | Standard in hospital and community PN care that included follow-up by a public health nurse post hospital discharge. |
| Mikami 2017 [38]  Brazil | Parallel RCT  2 arms  2009-2013 | Twins  88/83 | Face-to-face group 3AN (30 mins) not specified when by midwives. | Counselling on BF - importance of BF, how to prepare the nipples and breasts for BF, the main complications and difficulties and how to overcome them, BF positions for simultaneous feeding of twins and opportunity to ask questions. | 30 mins BF counselling by a hospital midwife brief guidance on initiating BF,  breast hygiene, colostrum/breastmilk, infants’ positions, duration of breastfeeding, frequency of feeds. |
| Morrow 1999 [39]  Mexico | Cluster  Parallel RCT  3 arms  1995-96 | 3 visits  52/34 | One-to-one, face-to-face, 1AN late pregnancy 2PN in weeks 1 and 2 by trained lay provider. Extra occasional visits also provided. | AN - benefits of EBF, anatomy and physiology, positioning and attachment, common myths, problems and solutions. PN - establishing BF, addressing concerns, providing information and social support. Key family members included. | No intervention. Mothers with BF problems were referred to own physicians. 50% born in a Baby Friendly accredited hospital. |
|  |  | 6 visits  44/34 | One-to-one, face-to-face, 1AN late pregnancy 4PN in weeks 1, 2, 4 and 8 by trained lay provider. Extra occasional visits also provided. | As above with additional PN visits |  |
| Muirhead 2006 [40]  Scotland | Parallel RCT  2 arms  1997-2002 | 112/113 | One-to-one, face-to-face, 1AN (plus more if requested) & PN every 2 days (or more) up to 28 days by peer supporters with training. | Breastfeeding problems encountered by peer support group women were solved where possible by the peer supporters directly during a visit or by telephone advice. The peer supporters were able to consult their supervising professional for additional help if required. | Women in both peer support and control groups received normal BF support (a community midwife for the first 10 days, health visitor after 10 days, BF support groups and breastfeeding workshops). |
| Nilsson 2017 [41]  Denmark | Cluster  Parallel RCT  2 arms  2013-2014 | 2,065 /1,476 | Face-to-face in hospital 3-5 PN days (number not specified) by nurses and midwives with extra training. | All mothers were orally introduced to the four core components - skin to skin, frequent feeding, good position and mother and father as equal partners with different roles in relation to BF (also highlighted on postcard). Communication based on Bandura's theory of self-efficacy with Kreuter’s theory of tailoring knowledge to the specific needs of the individual. BF counselling using manual and pamphlet. | Usual care provided by midwives. No Danish hospitals had UNICEF Baby Friendly accreditation at the time of the study. According to the national recommendations, HCPs are expected to base their breastfeeding support on the evidence-based national handbook on breastfeeding, but support varies across hospitals. |
| Ochola  2013 [42]  Kenya | Cluster  Parallel RCT  3 arms  2006-2008 | Facility  120/120 | One-to-one, face-to-face, 1AN (30-40mins) by researcher. | Counselling around benefits of EBF, preparation for BF, initiation and sustainability, position and attachment, prevention and management of BF challenges. | Usual general health and nutrition education which included and small amount of BF promotion. The health centre was not designated ‘Baby Friendly’ at the time of the study. |
|  |  | Home  120/120 | One-to-one, face-to-face, 1AN and 6PN, 1 in 1^st^ week and monthly until 5 months by peer counsellors with training. | Counselling around benefits of EBF, preparation for BF, initiation and sustainability, P&A, prevention and management of BF challenges - repeated at the additional counselling sessions depending on individual maternal needs as requested by the mothers. |  |
| Petrova 2009 [43]  USA | Parallel RCT  2 arms | Low income women  52/52 | One-to-one, face-to-face, 2AN and 5PN by telephone (15 mins) PN in hospital, and soon after discharge, end of 1st and 2nd week and 1st and 2nd months by lactation consultant. | AN - Benefits of BF, and encouragement to EBF and to delay introduction of formula. Women were told how frequently infants needed to be fed and for how long, and how to avoid breastfeeding associated discomfort and problems. PN - BF education and support by phone, encouraged to contact LC if experienced problems or complications. | Routine care - breastfeeding education and support during pregnancy and postpartum. Lactation consultant services were available for all postpartum women if breastfeeding problems arose during hospital stay. |
| Pound 2015 [44]  Canada | Parallel RCT  2 arms  2009-2012 | Jaundiced babies admitted to hospital  50/49 | One-to-one, face-to-face PN in hospital, and soon after discharge, weekly for 3 weeks (30 mins) planned but not all provided (unclear how many) by lactation consultant. | Based on established clinical practice guidelines and included: benefits of BF; assessment of the mother’s BF techniques, with correction as needed; taught how and when to use a breast pump and how to store breastmilk. Breast pumps and were provided at no cost for up to 6 weeks. Mother’s questions or concerns were addressed. Mother provided with a list of resources should further breastfeeding issues arise. | Standard care for jaundiced babies - formal standardized BF support. Mothers in both groups could consult private lactation consultants. |
| Pugh 2002 [45]  USA | Parallel RCT  2 arms  1999-2000 | Low income women  21/20 | One-to-one, face-to-face PN daily in hospital, home visits weeks 1, 2 and 4 and extra if needed, telephone support twice weekly to week 8 and weekly to month 6 by community health nurse/peer counselor team | In addition to usual care the intervention group received supplementary visits The community health nurse provided the mothers with professional knowledge, assessment skills, and educational support while simultaneously community-based peer counsellors share their personal breastfeeding experiences, empathize with the women’s situation, and serve as role models for successful BF. | Usual breastfeeding support, which consisted of support from hospital nurses, assistance by means of a telephone ‘‘warm line,’’ and one hospital visit by a lactation consultant if the participant delivered on a weekday. |
| Pugh 2010 [46]  USA | Parallel RCT  2 arms  2003-2005 | Low income women  168/160 | One-to-one, face-to-face, PN daily in hospital, x2 in 1st week, 3rd visit at 4 weeks (additional if required), then telephone every 2 weeks to 24 weeks, plus pager access 24/7 by health professional then peer supporter but with access to health professional. | Visits included strategies designed to strengthen maternal competence and commitment to BF; provide BF education; identify social support; ways to decrease fatigue and breast discomfort; links to community services to facilitate maintenance of BF. Education to facilitate successful BF, symptom management and problem solving for psychosocial issues. Infants weighed, measured, and professionally assessed. The calls consisted of discussing infant feeding, providing encouragement, assessing maternal wellbeing, and trouble-shooting potential problems. | Not designated UNICEF Baby Friendly. BF mothers admitted to each of the hospitals had access to an inpatient visit by a lactation consultant. After discharge, a hospital-based lactation consultant was available via a telephone “warm-line” (an answering machine checked at least every 24 hours). Once home, the participant could request an office visit with the lactation consultant. |
| Rasmussen 2011 [47]  USA | Parallel RCT  3 arms ( 2 relevant for this review)  2006-2007 | Women with high BMI  25/25 | One-to-one, face-to-face 1AN & PN 24 and 72 hrs after discharge by lactation consultants. | AN - asked questions about knowledge, expectations, and perceptions, answered questions, and reviewed practical points about BF. After delivery, nurses encouraged women in the targeted-care group to get up and move and asked visitors to leave the room if they had been there >2 hours or did not allow the mother privacy to BF. At 24 and 72 hours after discharge, received an additional call from a lactation consultant. Scripts were followed to standardize the assistance provided, but the lactation consultants asked questions and addressed issues as they deemed necessary. | Usual care- AN call was made to all participants but was less detailed for comparison group |
| Redman 1995 [48]  Australia | Parallel non-randomised (sequential selection), two arms  1989 | 115/120 | Face-to-face 1AN (3 hours) group at 28 weeks and 4PN one-to-one 1st in hospital, 2nd at 2-3 weeks after birth (telephone and home visit if required), 3rd at 6-8wks group, 4th telephone at 3 months, by registered nurse and midwife with BF qualifications | The aim of the breastfeeding programme (based on health belief model) was to increase women's confidence in their ability to BF and to encourage continuation of EBF for at least 4 months.  The advantages of BF; perceived problems with BF and strategies for overcoming these. Individual sessions were tailored to the needs of individual women. The group sessions offered the opportunity for social support. Modelling was used with demonstrations of BF and expressing. | Usual care - usual advice about BF from their doctor, the hospital staff and AN preparation classes. |
| Rojjanasrirat 1987 [49]  Australia | Two arm RCT but became a cluster RCT after two months as author randomized classes instead  1999-2000 | Working mothers  81/85 | 1AN (2-3hrs) 2PN 1st week and 4-6weeks by a certified lactation counsellor | AN = Breastfeeding education in a BF class that focused on preparing women to combine work and breastfeeding after childbirth. Strategies to involve the partner or significant other. An invited role model i.e. an experienced breastfeeding employed mother. PN calls - aimed to provide on-going assessment and support or help with problems identified, to reinforce the importance of BF information from AN class, and to establish a therapeutic relationship between the mother and the lactation consultant. | BF class - general information to initiate BF: advantages of breastmilk, anatomy of breasts; positioning, latching technique; pumping and milk storage (not specific for working mothers). Lecture format (2-3 hours) taught by various people. |
| Rossiter 1994 [50]  Australia | Parallel RCT  2 arms  1991-1992 | Vietnamese women in Australia  108/86 | Face-to-face group 3AN (2hr) (plus video) conducted in Vietnamese by the parenthood educators of the hospitals, with Vietnamese interpreter. | Education programme to: provide information on the benefits of BF in the Vietnamese language; relate this information to the women’s socioeconomic and cultural background; and discuss any misconceptions about the superiority of formula milk and the norm of infant feeding practices in Australia | Provided with the breastfeeding and childbirth pamphlets. |
| Rotheram-Fuller  2017 [51]  USA | Parallel RCT  2 arms  Year of study not provided. | 104/99 | Average of 14.9 PN contacts face-to-face or telephone (30 mins) by trained lay mentor mothers. | Each meeting was planned in a set sequence To identify: strengths or successes from the previous week; new topic for the day; how that topic was challenging for the mother; practice and problem solve how to address this challenge; review progress of the day; set a new goal. | Standard clinic care - not described. |
| Simonetti 2012 [52]  Italy | Parallel RCT  2 arms  2009 | 55/59 | One-to-one PN at least weekly for 6 weeks by telephone by midwife with UNICEF breastfeeding training. | During every phone call, the midwife gave support and all information on fully breastfeeding. | Standard counselling program, consisting of programmed visits with the physician at 1, 3 and 5 months after birth. Could call the midwife in case of breastfeeding problems. |
| Su  2007 [53]  Singapore | Parallel RCT  3 arms  2004-2005 | AN  150/151 | 1AN (15mins) by trained lactation counsellor (not stated if group or one-to-one). | One session AN BF education – video of benefits of BF, correct positioning, latch on, and breast care, and discussed common concerns. Given printed guides on breastfeeding and an opportunity to talk to a lactation counsellor for about 15 minutes. | Optional AN classes, which did address infant feeding, and PN visits by a lactation consultant should any problems with breastfeeding arise. |
|  |  | PN  148/151 | One-to-one face-to-face 2PN - 1st within 3 days in hospital, 2nd 1 or 2 weeks after birth by trained lactation counsellor. | Received hands-on instructions in latching on, proper positioning, and other techniques to avoid common complications. |  |
| Tahir  2013 [54]  Malaysia | Parallel RCT  2 arms  2010 | 179/178 | 12PN by telephone - 2 x monthly to 6 months by trained certified lactation counsellor. | Lactation counselling by telephone in addition to standard care (no further details provided). | Standard care - BF talks during immunisation, communication and BF advice with lactation counsellors or other health professionals during AN or PN follow ups. |
| Tuthill 2017 [55]  S.Africa | Parallel RCT  2 arms  2014 | HIV infected mothers  33/35 | One-to-one, face-to-face 1AN in 3rd trimester by trained HIV counsellor. | Exploring - feelings about pregnancy and decisions regarding infant feeding. Motivational interviewing techniques, including reflection, used to reduce ambivalence towards Information–Motivation–Behavioural Skills deficits. Used active listening to reflect back what she had been shared to stimulate further discussion surrounding concerns, questions and strategies to effectively initiate and maintain EBF. | Standard care - included a brief presentation by a counsellor or other health-care provider to a group prior to AN care. Content - AN and PN expectations, diet, delivery options and the promotion of EBF to all women, including those living with HIV. |
| Tylleskar 2011 [56]  Burkina Faso | Cluster  Parallel RCT  2 arms  2006-2008 | 392/402 | One-to-one, face-to-face 1AN and 4PN (at least) AN (3rd trimester), PN weeks 1, 2, 4, 8, 16 and 20 up to 6 months by trained peer supporters. | Peer counselling - given information about BF and peers provided support and addressed problems or referred women for specialist help. Mothers with any BF difficulties were referred to a health worker with training in lactation management. | Standard health care (not described). |
| Tylleskar  2011 [56]  S. Africa | Cluster Parallel RCT  2 arms  2006-2008 | 535/485 | One-to-one, face-to-face 1AN and 4PN (at least) - PN weeks 1, 4, 7 and 10 by trained peer supporters. | Peer counselling - given information about BF and peers provided support and addressed problems or referred women for specialist help. Mothers with any BF difficulties were referred to a public health provider who was aware of the mother’s HIV status. | The control clusters were visited by peer counsellors, with the same schedule as the intervention clusters, but they assisted families in obtaining birth certificates and social welfare grants. The peer counsellors for the intervention and control clusters were kept separate during the study. |
| Tylleskar 2011 [56]  Uganda | Cluster Parallel RCT  2 arms  2006-2008 | 396/369 | One-to-one, face-to-face 1AN and 4PN (at least) – PN weeks 1, 4, 7 and 10 by trained peer supporters. | Peer counselling - given information about BF and peers provided support and addressed problems or referred women for specialist help. Mothers with any BF difficulties were referred to health worker with training in lactation management. | Standard health care (not described). |
| Vidas 2011 [57]  Croatia | Parallel RCT  2 arms  2010 | 50/50 | The six basic exercises of autogenic training were taught face-to-face for 12 weeks in small groups to 10 members by trainer (not clear who) and mothers were encouraged to practice 3 times a day. | The 6 basic exercises of autogenic training were taught to promote self-confidence. Mothers encouraged to continue until 6 months. Authors state that “All such problems during BF mother will receive in a more peaceful way, with the support of professional staff and find the right solution to solve them. | Not described but advised to BF |
| Wilhelm 2015 [58]  USA | Parallel RCT  2 arms  2008 - 2010 | 26/27 | One-to-one, face-to-face 3PN, day 2, weeks 2 and 6 by researcher a health professional with training in motivational interviewing. | Motivational interviewing to increase self-efficacy by emphasizing personal choice. | Educational information about infant safety |
| Wong  2014 [59]  Hong Kong | Parallel RCT  2 arms  2013 | 233/236 | Face-to-face, AN (group 2hrs) one-to-one (10-15 mins) | BF education session based on WHO guidelines for Baby Friendly and evidence-based maternity care and 10-15mins to answer questions and address concerns. Handouts about the content discussed were distributed to participants at the end of the intervention and active communication with family and peers was encouraged. | Standard hospital antenatal care: routine maternal and fetal health checks by midwives or obstetricians along with health education to promote a healthy pregnancy. BF promoted and childbirth preparation and BF classes at no cost. |
| Zhu  2017 [60]  China | Parallel RCT  2 arms  2013-2014 | 180/172 | 2PN Group 2 days and 6 weeks, Telephone weekly until 6 weeks by trained obstetric nurse. | Programme aimed to increase knowledge and improve attitudes to BF, subjective norms and BF control (based on the Theory of Planned Behaviour). Included breastfeeding benefits, techniques and coping strategies. Individual instruction included: nutritional information, BF skills and dealing with breast pain, engorgement and other problems. Telephone counselling included emphasizing importance of BF, providing emotional support, coping strategies and dealing with problems. | Routine care included one AN BF education class, rooming-in, BF initiation within half an hour after childbirth, pamphlets on BF during hospital, and regular check-up and education on BF at 6 weeks, without telephone counselling. |

Note: RCT = randomised controlled trial, BF = breastfeeding, EBF= Exclusive breastfeeding, AN= antenatal, PN=postnatal, BMI = body mass index

**References**

1. Agrasada GV, Gustafsson J, Kylberg E, Ewald U: **Postnatal peer counselling on exclusive breastfeeding of low-birthweight infants: a randomized, controlled trial** *Acta Paediatrica* 2005, **94**:1109-1115.

2. Ahmadi S, Kazemi F, Masoumi SZ, Parsa P, Roshanaei G: **Intervention based on BASNEF model increases exclusive breastfeeding in preterm infants in Iran: a randomized controlled trial**. *International Breastfeeding Journal* 2016, **11**:30.

3. Aidam BA, Perez-Escamilla R, Lartey A: **Lactation counseling increases exclusive breast-feeding rates in Ghana** *The Journal of Nutrition* 2005, **135**:1691-1695.

4. Aksu H, Kucuk M, Duzgun G: **The effect of postnatal breastfeeding education/support offered at home 3 days after delivery on breastfeeding duration and knowledge: a randomized trial**. *The Journal of Maternal-Fetal & Neonatal Medicine* 2011, **24**:354-361.

5. Anderson AK, Damio G, Young S, Chapman DJ, Perez-Escamilla R: **A randomized trial assessing the efficacy of peer counseling on exclusive breastfeeding in a predominantly Latina low-income community**. *JAMA Pediatrics* 2005, **159**:836-841.

6. Ara G, Khanam M, Papri N, Kabir I, Dibley M: **Does peer counseling promote appropriate infant feeding and better growth in infants in urban slums in Bangladesh?** . *Annals of Nutrition and Metabolism* 2017 **71 (Supplement 2)**:397-398.

7. Bonuck K, Stuebe A, Barnett J, Fletcher J, Bernstein P: **Routine, primary-care based interventions to increase breastfeeding: Results of two randomized clinical trials**. *Breastfeeding Medicine* 2013, **8**:S-19.

8. Bonuck K, Freeman K, Trombley M: **Randomized controlled trial of a prenatal and postnatal lactation consultant intervention on infant health care use**. *Archives of Pediatric and Adolescent Medicine* 2006, **160**:953-960.

9. Brent NB, Redd B, Dworetz A, D'Amico F, Greenberg JJ: **Breast-feeding in a low-income population. Program to increase incidence and duration** In: *Archives of Pediatrics & Adolescent Medicine* vol. 149; 1995 798-803.

10. Cangol E, Sahin NH: **The effect of a breastfeeding motivation program maintained during pregnancy on supporting breastfeeding: A randomized controlled trial**. *Breastfeeding Medicine* 2017, **12**(4):218-226.

11. Carlsen EM, Kyhnaeb A, Renault KM, Cortes D, Michaelsen KF, Pryds O: **Telephone-based support prolongs breastfeeding duration in obese women: a randomized trial**. *The American Journal of Clinical Nutrition* 2013, **98**:1226-1232.

12. Chan MY, Ip WY, Choi KC: **The effect of a self-efficacy-based educational programme on maternal breastfeeding self-efficacy, breastfeeding duration and exclusive breastfeeding rates: A longitudinal study** *Midwifery* 2016 **36**:92-98.

13. Chapman D, Damio G, Young S, Perez-Escamilla R: **Effectiveness of breastfeeding peer counseling in a low-income, predominantly Latina population: a randomized controlled trial**. *Archives of Pediatric and Adolescent Medicine* 2004, **158**:897-902.

14. Chapman DJ, Morel K, Bermúdez-Millán A, Young S, Damio G, Pérez-Escamilla R: **Breastfeeding education and support trial for overweight and obese women: a randomized trial**. *Pediatrics* 2013, **131**(1):e162-170.

15. Ciftci EK, Arikan D: **The effect of training administered to working mothers on maternal anxiety levels and breastfeeding habits**. *Journal of Clinical Nursing* 2012, **21**:2170-2178.

16. Coutinho SB, Lira PI, Carvalho Lima M, Ashworth A: **Comparison of the effect of two systems for the promotion of exclusive breastfeeding**. *The Lancet* 2005, **366**(9491):1094-1100.

17. Davies-Adetugbo AA, Adetugbo K, Orewole Y, Fabiyi AK: **Breast-feeding promotion in a diarrhoea programme in rural communities**. *Journal of Diarrhoeal Diseases Research* 1997, **15**:161-166.

18. Dennis C-L, Hodnett E, Gallop R, Chalmers B: **The effect of peer support on breast-feeding duration among primiparous women: a randomized controlled trial** *Canadian Medical Association Journal* 2002 **166**:21-28.

19. de Oliveira LD, Giugliani ERJ, do Espirito Santo LC, Franca MCT, Weigert EML, Kohler CVF, de Lourenzi Bonilha AL: **Effect of intervention to improve breastfeeding technique on the frequency of exclusive breastfeeding and lactation-related problems**. *Journal of Human Lactation* 2006, **22**:315-321.

20. Di Napoli A, Di Lallo D, Fortes C, Franceschelli C, Armeni E, Guasticchi G: **Home breastfeeding support by health professionals: findings of a randomized controlled trial in a population of Italian women**. *Acta Paediatrica* 2004, **93**:1108-1114.

21. Edwards RC, Thullen MJ, Korfmacher J, Lantos JD, Henson LG, Hans SL: **Breastfeeding and complementary food: randomized trial of community doula home visiting** *Pediatrics* 2013 **132 (Suppl 2)**:S160-166.

22. Elliott-Rudder M, Pilotto L, McIntyre E, Ramanathan S: **Motivational interviewing improves exclusive breastfeeding in an Australian randomised controlled trial** *Acta Paediatrica* 2014 **103**:e11-16.

23. Fu ICY, Fong DYT, Heys M, Lee ILY, Sham A, Tarrant M: **Professional breastfeeding support for first-time mothers: a multicentre cluster randomised controlled trial**. *British Journal of Obstetrics and Gynaecology* 2014, **121**:1673-1683.

24. Gijsbers B, Mesters I, Knottnerus JA, Kester ADM, Van Schayck CP: **The success of an educational program to promote exclusive breastfeeding for 6 months in families with a history of asthma: A randomized controlled trial** *Pediatric Asthma, Allergy and Immunology* 2006 **19**(4):214-222.

25. Graffy J, Taylor J, Williams A, Eldridge S: **Randomised controlled trial of support from volunteer counsellors for mothers considering breastfeeding** *British Medical Journal* 2004 **328**:26.

26. Grossman LK, Harter C, Sachs L, Kay A: **The effect of postpartum lactation counseling on the duration of breast-feeding in low-income women**. *American Journal of Diseases of Children* 1990, **144**:471-474.

27. Haider R, Ashworth A, Kabir I, Huttly SR: **Effect of community-based peer counsellors on exclusive breastfeeding practices in Dhaka, Bangladesh: a randomised controlled trial**. *The Lancet* 2000, **356**:1643-1647.

28. Jones DA, West RR: **Effect of a lactation nurse on the success of breast-feeding: a randomised controlled trial**. *Journal of Epidemiology & Community Health* 1986 **40**:45-49.

29. Khresheh R, Suhaimat A, Jalamdeh F, Barclay L: **The effect of a postnatal education and support program on breastfeeding among primiparous women: a randomized controlled trial**. *International Journal of Nursing Studies* 2011, **48**(9):1058-1065.

30. Kimani-Murage EW, Griffiths PL, Wekesah FM, Wanjohi M, Muhia N, Muriuki P *et al*: **Effectiveness of home-based nutritional counselling and support on exclusive breastfeeding in urban poor settings in Nairobi: a cluster randomized controlled trial**. *Globalization and Health* 2017 **13**:90.

31. Kronborg H, Vaeth M, Olsen J, Iversen L, Harder I: **Effect of early postnatal breastfeeding support: a cluster-randomized community based trial** *Acta Paediatrica* 2007 **96**:1064-1070.

32. Kupratakul J: **A randomized controlled trial of knowledge sharing practice with empowerment strategies in pregnant women to improve exclusive breastfeeding during the first six months postpartum** *Doctoral Dissertation.* Chulalongkorn Unversirty; 2010

33. Lynch SA, Koch AM, Hislop TG, Coldman AJ: **Evaluating the effect of a breastfeeding consultant on the duration of breastfeeding**. *Canadian Journal of Public Health* 1986, **77**:190-195.

34. Mattar CN, Chong Y-S, Chan Y-S, Chew A, Tan P, Chan Y-H, Rauff MH-J: **Simple antenatal preparation to improve breastfeeding practice: a randomized controlled trial** *Obstetrics & Gynecology* 2007 **109**:73-80.

35. McDonald SJ, Henderson JJ, Faulkner S, Evans SF, Hagan R: **Effect of an extended midwifery postnatal support programme on the duration of breastfeeding: a randomised controlled trial**. *Midwifery* 2010, **26**:88-100.

36. McLachlan HL, Forster DA, Amir LH, Cullinane M, Shafiei T, Watson LF, Ridgway L, Cramer RL, Small R: **Supporting breastfeeding in local communities (SILC) in Victoria, Australia: a cluster randomised controlled trial**. *BMJ Open* 2016, **6**:e008292.

37. McQueen KA, Dennis CL, Stremler R, Norman CD: **A pilot randomized controlled trial of a breastfeeding self-efficacy intervention with primiparous mothers** *Journal of Obstetric, Gynecologic, and Neonatal Nursing: JOGNN* 2011 **40**(1):35-46.

38. Mikami FCF, de Lourdes Brizot M, Tase TH, Saccuman E, Vieira Francisco RP, Zugaib M: **Effect of prenatal counseling on breastfeeding rates in mothers of twins**. *Journal of Obstetric, Gynecologic & Neonatal Nursing* 2017, **46**:229-237.

39. Morrow AL, Guerrero ML, Shults J, Calva JJ, Lutter C, Bravo J, Ruiz-Palacios G, Morrow RC, Butterfoss FD: **Efficacy of home-based peer counselling to promote exclusive breastfeeding: a randomised controlled trial**. *The Lancet* 1999, **353**:1226-1223.

40. Muirhead PE, Butcher G, Rankin J, Munley A: **The effect of a programme of organised and supervised peer support on the initiation and duration of breastfeeding: a randomised trial** *British Journal of General Practice* 2006 **56**:191-197.

41. Nilsson IMS, Strandberg-Larsen K, Knight CH, Hansen AV, Kronborg H: **Focused breastfeeding counselling improves short- and long-term success in an early-discharge setting: A cluster-randomized study**. *Maternal and Child Nutrition* 2017, **13**:e12432.

42. Ochola SA, Labadarios D, Nduati RW: **Impact of counselling on exclusive breast-feeding practices in a poor urban setting in Kenya: a randomized controlled trial**. *Public Health Nutrition* 2013, **16**:1732-1740.

43. Petrova A, Ayers C, Stechna S, Gerling JA, Mehta R: **Effectiveness of exclusive breastfeeding promotion in low-income mothers: a randomized controlled study**. *Breastfeeding Medicine* 2009 **4**(2):63-69.

44. Pound CM, Moreau K, Rohde K, Barrowman N, Aglipay M, Farion KJ, Plint AC: **Lactation support and breastfeeding duration in jaundiced infants: a randomized controlled trial**. *Plos One* 2015, **10**(3):e0119624.

45. Pugh LC, Milligan RA, Frick KD, Spatz D, Bronner Y: **Breastfeeding duration, costs, and benefits of a support program for low-income breastfeeding women**. *Birth: Issues in Perinatal Care* 2002 **29**(2):95-100.

46. Pugh LC, Serwint JR, Frick KD, Nanda JP, Sharps PW, Spatz DL, Milligan RA: **A randomized controlled community-based trial to improve breastfeeding rates among urban low-income mothers**. *Academic Pediatrics* 2010 **10**(1):14-20.

47. Rasmussen KM, Dieterich CM, Zelek ST, Altabet JD, Kjolhede CL: **Interventions to increase the duration of breastfeeding in obese mothers: the Bassett Improving Breastfeeding Study**. *Breastfeeding Medicine* 2011, **6**(2):69-75.

48. Redman S, Watkins J, Evans L, Lloyd D: **Evaluation of an Australian intervention to encourage breastfeeding in primiparous women**. *Health Promotion International* 1995, **10**(2):101-113.

49. Rojjanasrirat W: **The effects of a nursing intervention on breastfeeding duration among primiparous mothers planning to return to work**. *Doctoral Dissertation.* Kansas: University of Kansas; 2000.

50. Rossiter JC: **The effect of a culture-specific education program to promote breastfeeding among Vietnamese women in Sydney**. *International Journal of Nursing Studies* 1994, **31**:369-379.

51. Rotheram-Fuller EJ, Swendeman D, Becker KD, Daleiden E, Chorpita B, Harris DM, Mercer NT, Rotheram-Borus MJ: **Replicating Evidence-Based Practices with Flexibility for Perinatal Home Visiting by Paraprofessionals** *Maternal & Child Health Journal* 2017 **21**:2209-2218, 2017 Dec.

52. Simonetti V, Palma E, Giglio A, Mohn A, Cicolini G: **A structured telephonic counselling to promote the exclusive breastfeeding of healthy babies aged zero to six months: a pilot study**. *International Journal of Nursing Practice* 2012, **18**:289-294.

53. Su LL, Chong YS, Chan YH, Chan YS, Fok D, Tun KT, Ng FSP, Rauff M: **Antenatal education and postnatal support strategies for improving rates of exclusive breastfeeding: Randomised controlled trial**. *British Medical Journal* 2007, **335**(7620):596-599.

54. Tahir NM, Al-Sadat N: **Does telephone lactation counselling improve breastfeeding practices? A randomised controlled trial**. *International Journal of Nursing Studies* 2013, **50**:16-25.

55. Tuthill EL, Butler LM, Pellowski JA, McGrath JM, Cusson RM, Gable RK, Fisher JD: **Exclusive breast-feeding promotion among HIV-infected women in South Africa: an Information-Motivation-Behavioural Skills model-based pilot intervention** *Public Health Nutrition* 2017 **20**:1481-1490.

56. Tylleskar T, Jackson D, Meda N, Engebretsen IMS, Chopra M, Diallo AH *et al*: **Exclusive breastfeeding promotion by peer counsellors in sub-Saharan Africa (PROMISE-EBF): a cluster-randomised trial**. *The Lancet* 2011, **378**:420-427.

57. Vidas M, Folnegovic-Smalc V, Catipovic M, Kisic M: **The application of autogenic training in counseling center for mother and child in order to promote breastfeeding**. *Collegium Antropologicum* 2011, **35**:723-731.

58. Wilhelm SL, Aguirre TM, Koehler AE, Rodehorst TK: **Evaluating motivational interviewing to promote breastfeeding by rural Mexican-American mothers: the challenge of attrition** *Issues in Comprehensive and Pediatric Nursing* 2015 **38**:7-21.

59. Wong KL, Fong DYT, Lee ILY, Chu S, Tarrant M: **Antenatal education to increase exclusive breastfeeding: a randomized controlled trial** *Obstetrics and Gynecology* 2014 **5**:961-968.

60. Zhu Y, Zhang Z, Ling Y, Wan H: **Impact of intervention on breastfeeding outcomes and determinants based on theory of planned behavior**. *Women & Birth* 2017, **30**:146-152.
